# Eukaryotic Initiation Factor 3F (eIF3F) Regulates the IRES-Mediated Translation of Bcl-xL via Its Interaction with Programmed Cell Death 4 (PDCD4) Protein

**DOI:** 10.3390/ijms27093955

**Published:** 2026-04-29

**Authors:** Veda Hegde, Divya K. Sharma, Harshil Patel, Pavan Lakshmi Narasimha, Jason Luddu, Rebecca Mubaya, Martin Holcik, Nehal Thakor

**Affiliations:** 1 Department of Biological Sciences, University of Lethbridge, Lethbridge, AB T1K 3M4, Canada; veda.hegde@uleth.ca (V.H.); divyaskhandige@gmail.com (D.K.S.); p.lakshminarasimha@uleth.ca (P.L.N.); jason.luddu200@gmail.com (J.L.); rebecca.mubaya@uleth.ca (R.M.); 2 Department of Health Sciences, Carleton University, Ottawa, ON K1S 5B6, Canada; martin.holcik@carleton.ca; 3 Arnie Charbonneau Cancer Institute, Cumming School of Medicine, University of Calgary, Calgary, AB T2N 1N4, Canada

**Keywords:** PDCD4, eIF3, mRNA translation, IRES-mediated translation, ITAFs, Bcl-xL

## Abstract

Programmed cell death 4 (PDCD4) protein is a tumour suppressor protein that inhibits mRNA translation by inhibiting RNA helicase, eukaryotic initiation factor 4A (eIF4A). We have previously reported that PDCD4 interacts with the internal ribosome entry site (IRES) element of B-cell lymphoma extra-large (Bcl-xL) mRNA and inhibits its IRES-mediated translation initiation. S6 kinase (S6K)-mediated phosphorylation of PDCD4 activates its degradation and derepresses IRES-mediated translation initiation of Bcl-xL mRNA. eIF3F (one of the subunits of eIF3 complex) was reported to recruit S6K to phosphorylate eIF3G. Therefore, we investigated the possibility of co-regulation of PDCD4 and eIF3F by S6K and the regulation of IRES-mediated translation initiation by PDCD4–eIF3F. Here, we demonstrated that PDCD4 interacts with several subunits of eIF3. Specifically, eIF3F directly interacts with PDCD4 in an RNA-independent manner. Depletion of PDCD4 in glioblastoma (GBM) cells resulted in decreased levels of certain eIF3 subunits, including eIF3F. Additionally, depletion of eIF3F from GBM cells decreased the levels of PDCD4 protein. We also showed that PDCD4 and eIF3F directly interact with Bcl-xL RNA independently of each other. By performing IRES reporter, polysome profiling assays and EMSA we have demonstrated that eIF3F regulates IRES-mediated translation of Bcl-xL mRNA, likely via its interaction with PDCD4.

## 1. Introduction

A significant body of work indicates that the regulation of the cellular internal ribosome entry site (IRES)-mediated translation can be influenced by IRES trans-acting factors (ITAFs) [1,2,3]. ITAFs are known to affect the IRES structures of specific mRNAs, altering their affinity for ribosomes and translation initiation factors [1,4,5]. Each ITAF has the capability to either positively or negatively regulate translation [2]. ITAFs modulate the IRES-mediated translation initiation by interacting with the ribosome or other proteins (such as eukaryotic initiation factors), changing the mRNA conformation upon binding, and serving as RNA chaperones [1,4,6,7]. The protein levels, post-translational modifications, and availability of eIFs and ITAFs are critical for modulating the IRES-mediated translation initiation [1,8,9].

PDCD4 is a well-established inhibitor of mRNA translation [10,11]. As such, PDCD4 binds to eIF4A, which results in the loss of eIF4A’s helicase activity and ultimately inhibition of canonical translation initiation [12,13]. Additionally, PDCD4 is reported to inhibit canonical mRNA translation by binding to the poly(A)-binding protein (PABP) [14], and eIF4G [15,16]. However, our previous work demonstrated that PDCD4 can also act as an ITAF [17] and negatively regulates IRES-mediated translation by directly binding to IRES elements of Bcl-xL and the X-linked inhibitor of apoptosis protein (XIAP) [17].

The mammalian target of rapamycin (mTOR)/ribosomal protein S6 kinase 1 and 2 (S6K1 and S6K2) axis regulates PDCD4 by phosphorylating PDCD4 at serine 67(S67), leading to its proteasomal degradation [17,18,19]. The degradation of PDCD4 enhances the activity of canonical translation initiation, and also the IRES-mediated translation of XIAP and Bcl-xL mRNAs is upregulated in HEK293T cells [12,17,19].

Like for PDCD4, the mTOR/S6K1/S6K2 axis has been shown to regulate the activity of eIF3 [17,18,19]. As such, S6K1 physically interacts with eIF3F, and to a lesser extent with eIF3G, which leads to the phosphorylation of eIF3 [20]. This, in turn, enhances the mRNA translation [20]. Further, we and others have shown that eIF3 also regulates the translation initiation of IRES-containing mRNAs such as XIAP and HCV [21,22]. A recent publication has shown that PDCD4 interacts with eIF3G and eIF3I, when it is bound to the mRNA channel on 40S ribosome [16], suggesting that PDCD4–eIF3 (or some of its subunits) complex plays a crucial role in mRNA translation regulation. As both PDCD4 and eIF3 regulate IRES-mediated translation initiation and both are controlled by a common mTOR/S6K axis, we were interested in defining their joint role in IRES-mediated translation of a cellular mRNA.

In this study, we show that PDCD4 interacts with 10 subunits of eIF3. Notably, PDCD4 interacts directly with eIF3F in an RNA-independent manner, and they regulate each other’s stability. However, to our surprise, mTOR/S6K signalling did not affect their interaction. Depletion of eIF3F (part of module three of eIF3) destabilizes PDCD4 and enhances Bcl-xL IRES-mediated translation. In contrast, depletion of eIF3G (a known direct interaction partner of PDCD4; module one of eIF3) or eIF3D (part of module two of eIF3) modestly affected the levels of PDCD4 but did not significantly affect the levels of Bcl-xL protein. We also show that individually PDCD4 and eIF3F interacts directly with Bcl-xL IRES RNA. Our data suggests that PDCD4 and eIF3F (but not eIF3G or eIF3D) work as a complex in regulating Bcl-xL expression. In summary, PDCD4 and eIF3F work together in regulating IRES-mediated translation of Bcl-xL and the mTOR/S6K1&2 axis does not regulate the interaction between eIF3F and PDCD4. Interestingly, PDCD4–eIF3F-dependent regulation of Bcl-xL is observed in the tested glioblastoma cells, including brain tumour stem cells (BTSCs). However, non-cancer cells WI-38 (fibroblasts) do not show similar protein regulation. Therefore, we propose that PDCD4–eIF3F-dependent regulation of Bcl-xL is specifically relevant to GBM pathophysiology.

## 2. Results

### 2.1. eIF3F and PDCD4 Interact with Each Other Directly in an RNA-Independent Manner

**Figure 1:**
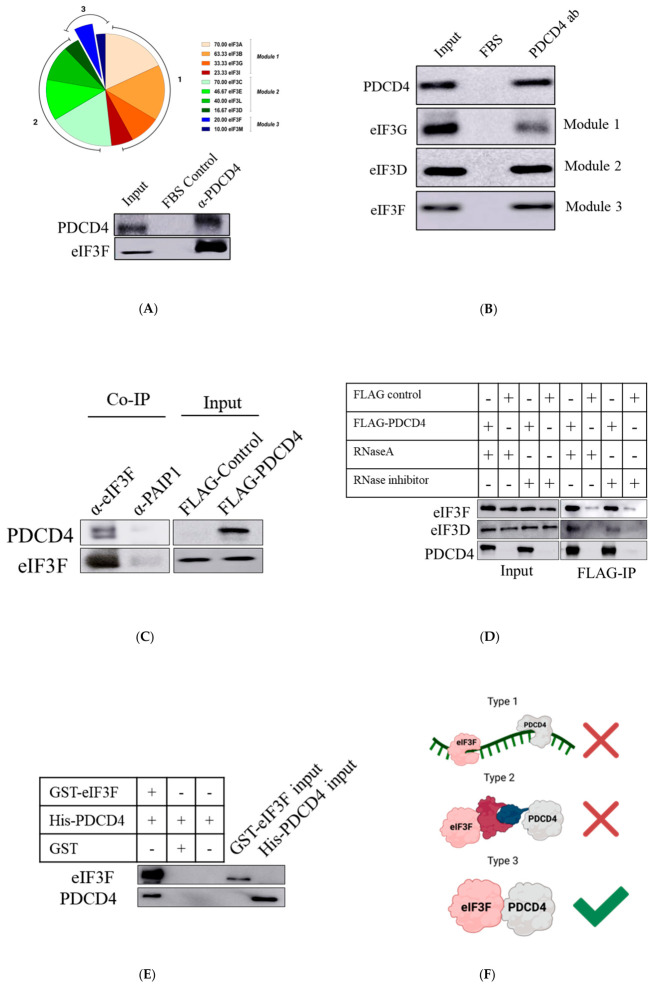
eIF3F and PDCD4 interact in an RNA-independent manner: (**A**; **top** panel) PDCD4 protein interacts with 10 eIF3 subunits as revealed by IP-MS experiment. eIF3F interaction is represented as an exploded slice and consists of a fold change of 20.00 with respect to FBS negative control. (**A**; **bottom** panel) Western blot analysis of the IP-MS samples confirms that endogenous eIF3F interacts with endogenous PDCD4 in the absence of crosslinking agent; (**B**) Western blot analysis confirms the endogenous interaction of one subunit from each major module of eIF3 (eIF3F, eIF3G, and eIF3D) protein with PDCD4; (**C**) reciprocal IP was performed using the eIF3F antibody. (**D**) Co-immunoprecipitated FLAG-PDCD4 along with eIF3F as seen in the IP lane; eIF3F and eIF3D were co-immunoprecipitated with FLAG-PDCD4 (right panel, lanes 1 and 3); there was no difference in their interaction between RNase A treatment and RNase inhibitor treatment.; (**E**) the prey protein recombinant PDCD4 binds directly to the bait protein recombinant eIF3F. PDCD4 is pulled down with eIF3F.; (**F**) PDCD4 and eIF3F can interact in one of the following ways: PDCD4 and eIF3F may interact with the same RNA molecule, PDCD4 and eIF3F may be part of the same protein complex, or there could be direct interaction between PDCD4 and eIF3F. (**F**) was created by VH using BioRender^TM^.

Individually, both PDCD4 and eIF3 are reported to regulate IRES-mediated translation [17,22]. A recent finding showed that PDCD4 interacts directly with eIF3G and eIF3I (subunits of eIF3 complex) when PDCD4 occupies the mRNA channel on the 40S ribosome [16]. In order to understand the role of PDCD4–eIF3 interaction in IRES-mediated translation regulation, we first determined if PDCD4 interacts with other eIF3 subunits. To this end, we employed immunoprecipitation coupled with mass spectrometry (IP-MS) analysis. In this experiment, we pulled down endogenous PDCD4 (from U343 cells) without crosslinking to capture dynamic interaction partners of PDCD4 in basal conditions. We identified 10 (eIF3A, eIF3B, eIF3C, eIF3D, eIF3E, eIF3F, eIF3G, eIF3I, eIF3L, and eIF3M) eIF3 subunits interacting with PDCD4, suggesting that PDCD4 interacts with eIF3 complex under basal growth conditions (Figure 1A, top panel). We also identified poly(A)-binding protein (PABP), eIF4G, and eIF4A (known interaction partners of PDCD4) in MS hits (Supplemental Table S3). By performing a Western blot of the same sample submitted for MS analysis, we validated PDCD4–eIF3F interaction (Figure 1A, bottom panel).

To further confirm the interaction between PDCD4 and eIF3 subunits, we performed endogenous immunoprecipitation (Endo-IP) in U343 glioblastoma cells. Here, PDCD4 antibody-coated beads were used to pull down the eIF3 subunits that interact with it. Qualitative analysis by Western blot confirmed the pull-down of one eIF3 subunit from the three major modules of eIF3 (Figure 1B). Input lanes show the presence of all the respective proteins (PDCD4, eIF3F, eIF3G, and eIF3D) in the cell lysates. The FBS lanes serve as the negative control, where the beads were coated with FBS instead of PDCD4 antibody. The absence of bands in the FBS lanes confirms the lack of non-specific interaction of the cell lysate proteins directly with the beads. The presence of bands in the IP lane confirms the interaction of PDCD4 with eIF3F (module three), eIF3G (module one), and eIF3D (module two).

We have previously demonstrated that PDCD4 regulates IRES-mediated translation of Bcl-xL and that S6K-mediated phosphorylation of PDCD4 plays a critical role in this regulation [17]. In a separate study, Martineau et al. showed that S6K directly interacts with eIF3F [20]. As both eIF3F and PDCD4 are direct interaction partners of S6K, we focused our study on characterizing the interaction between PDCD4 and eIF3F and hypothesized a co-regulation of the PDCD4–eIF3F complex by S6K. Therefore, first, we validated the interaction between PDCD4 and eIF3F. To this end, a reciprocal IP was conducted using the eIF3F antibody. Subsequent Western blot analysis with a conformation-specific secondary anti-rabbit antibody raised in mice confirmed co-immunoprecipitation of PDCD4 with the eIF3F subunit (Figure 1C). Interestingly, an additional band, potentially representing the FLAG-tagged or post-translationally modified form of PDCD4, was observed along with PDCD4 in the eIF3F immunoprecipitated sample (Figure 1C). Notably, PDCD4 was not detected in the PAIP1 IP sample (negative control), confirming an interaction of PDCD4 with eIF3F (Figure 1C).

To examine if PDCD4–eIF3F interaction is RNA-dependent or not, two separate FLAG IP experiments were performed either in the presence of RNase A enzyme (use of RNase inhibitor was avoided) or in the presence of RNase inhibitor (RNase A enzyme was not used). Both eIF3F and eIF3D were co-immunoprecipitated with FLAG-PDCD4 in both RNase A-treated or RNase-inhibited cell lysates (Figure 1D). The protein levels of eIF3F and eIF3D were found to be similar in all four lysates, as seen in the input blot. Endogenous PDCD4 protein was not detected in the FLAG tag control lysate due to inherently low levels of PDCD4 in U343 cells. Additionally, there was no significant interaction of eIF3F or eIF3D subunits with the FLAG-tag (negative control lanes). Therefore, we conclude that the PDCD4–eIF3F and PDCD4–eIF3D interactions are RNA-independent (Figure 1D). However, although PDCD4 and eIF3F interaction is not RNA-dependent, we cannot exclude the possibility that RNA that might be bound to PDCD4, eIF3F, and/or eIF3D may be protected from RNase degradation.

To investigate if PDCD4 interacts directly with eIF3F, in vitro protein pull-down was performed using glutathione Sepharose matrix. As such, bait protein GST-eIF3F was captured on the GST matrix. After stringent washing steps, the prey protein His-PDCD4 was incubated with the bait protein captured on the affinity matrix. After further washing steps, Western blot analysis was performed using eIF3F and PDCD4 antibodies. His-PDCD4 was detected in the presence of GST-eIF3F but not with GST or glutathione Sepharose matrix only, suggesting a direct protein–protein interaction between eIF3F and PDCD4 (Figure 1E).

As summarized in Figure 1F, the interaction of eIF3F and PDCD4 proteins demonstrated by co-IP can be a result of three major types of protein association. In type 1, PDCD4 and eIF3 may interact with the same RNA independently of each other. However, through the RNase A treatment, we have shown that eIF3F–PDCD4 interaction is RNA-independent. In Type 2, PDCD4 and eIF3F (one among many eIF3 subunits) may participate in a shared protein complex, including additional proteins, consequently leading to their co-immunoprecipitation. This implies the likelihood of a physical association between PDCD4 and eIF3F within the context of a common protein complex. However, this type of interaction was ruled out by performing an in vitro pull-down assay with purified components. In type 3 interaction, PDCD4 and the eIF3F subunit physically interact with each other directly (Figure 1F). Our data support this interaction by showing that PDCD4 and eIF3F directly interact with each other in the absence of additional proteins or mRNA.

### 2.2. S6K Activity Does Not Impact the Interaction Between PDCD4 and eIF3F

**Figure 2:**
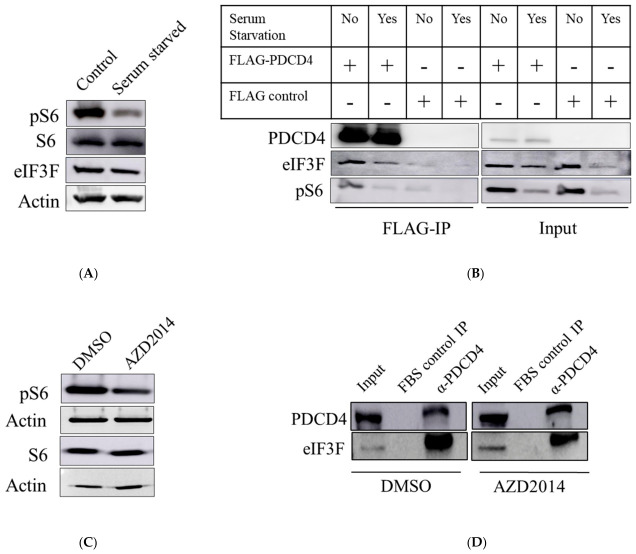
mTOR/S6K axis does not affect eIF3F–PDCD4 interaction: (**A**) serum starvation decreased the S6 phosphorylation, which suggests decreased S6K activity; (**B**) eIF3F and pS6 were co-immunoprecipitated with FLAG-PDCD4. This interaction decreased with serum starvation, seen in the co-IP lanes. However, the input lanes indicated a decrease in eIF3F and pS6 protein levels in the total cell lysate. This suggests that the PDCD4–eIF3F interaction is not affected by serum starvation; (**C**) U343 cells were treated with DMSO and a sub-lethal dose of mTOR inhibitor AZD2014. AZD2014 inhibitor activity was confirmed by observing the levels of S6 and pS6; (**D**) endogenous eIF3F is co-immunoprecipitated along with endogenous PDCD4 and AZD2014 treatment did not affect endogenous PDCD4–eIF3F interaction.

S6K1 is known to interact with eIF3F and to regulate the activity of eIF3 [20]. Moreover, both S6K1 and S6K2 were reported to regulate the levels of PDCD4 via the proteasomal degradation pathway [23]. As both PDCD4 and eIF3F are known to interact with S6K, we wanted to investigate if S6K1 and 2 play any role in the interaction between eIF3F and PDCD4. To this end, we employed S6K double knockout MEFs. First, we performed Western blot analysis of the phosphorylated form of S6 protein (the physiological target of S6Ks) in wildtype and S6K double knockout cells (Supplemental Figure S1A). The complete lack of phospho-S6 (pS6) confirms that S6K1 and 2 activity was absent in the S6K double knockout cells (Supplemental Figure S1A). To examine if PDCD4 and eIF3F interact in the S6K1 and 2 double knockout conditions, FLAG-PDCD4 was expressed in S6K1 and 2 expressing MEFs (wildtype) and S6K1 and 2 double knockout MEFs (Supplemental Figure S1B). Subsequently, we performed FLAG co-IP followed by Western blot analysis. The membrane was probed with anti-PDCD4, anti-eIF3F, anti-S6, and anti-pS6 antibodies. There were no significant changes in the level of co-immunoprecipitated eIF3F in FLAG-PDCD4 and FLAG-tag expressed MEFs (Supplemental Figure S1B). Unlike U343, glioblastoma cells (Figure 2B), we did not observe interaction between FLAG-PDCD4 and eIF3F in MEFs (Supplemental Figure S1B). Therefore, the MEFs model may not be suitable for studying the effect of S6K1 and 2 on the interaction between PDCD4 and eIF3F. Accordingly, we investigated the role of S6K1/2 in PDCD4–eIF3F interaction in U343 cells. To this end, we serum-deprived U343 cells for 24 h and performed Western blot analysis to examine the levels of pS6. A reduced phosphorylation of S6 was observed in U343 cells upon serum starvation (Figure 2A). The expression of S6 protein remained unaffected during serum starvation (Figure 2A). As expected, these observations suggest that serum deprivation inhibits the activity of S6K1/2 in U343 cells (Figure 2A). Subsequently, we expressed FLAG-PDCD4 in U343 cells and performed FLAG co-IP experiments in control and serum-deprived U343 cells (Figure 2B and Figure S1C). eIF3F and pS6 were found to be co-immunoprecipitated with FLAG-PDCD4 (Figure 2B and Figure S1C). The eIF3F to PDCD4 ratio was found to be decreased by about 25% in serum-starved conditions when normalized with the amount of PDCD4 immunoprecipitated (Supplemental Figure S1D). However, there was also a similar decrease in the eIF3F levels upon serum starvation in input (cell lysate) lanes (Figure 2B and Figure S1C). The observed reduction in PDCD4–eIF3F interaction may therefore be attributed to a decreased eIF3F protein level rather than a variation in the PDCD4–eIF3F interaction (Figure 2B and Figure S1C). Reduced phosphorylation of S6 protein confirms that serum starvation indeed inhibited S6K1/2 (Figure 2B and Figure S1C).

The influence of mTOR is important for the activation of S6K1/2. Accordingly, we treated U343 cells with a sub-lethal dose of mTOR inhibitor (AZD2014) and monitored the phosphorylation of S6 protein. As expected, AZD2014 treatment decreased the phosphorylation of S6 protein but did not reduce the levels of S6 protein (Figure 2C). We performed an Endo-IP experiment in DMSO (control)- and AZD2014-treated U343 cells using antibodies against PDCD4 (Figure 2D). FBS was used as a negative control for IP. Endogenous eIF3F was co-immunoprecipitated with endogenous PDCD4 under DMSO (control) treatment conditions. This endogenous interaction was not affected by AZD2014 treatment (Figure 2D). Collectively, these data suggest that mTOR/S6K did not measurably change interaction between PDCD4 and eIF3F under these tested conditions.

### 2.3. PDCD4 and eIF3F Regulate Each Other’s Levels and Bcl-xL Levels

**Figure 3:**
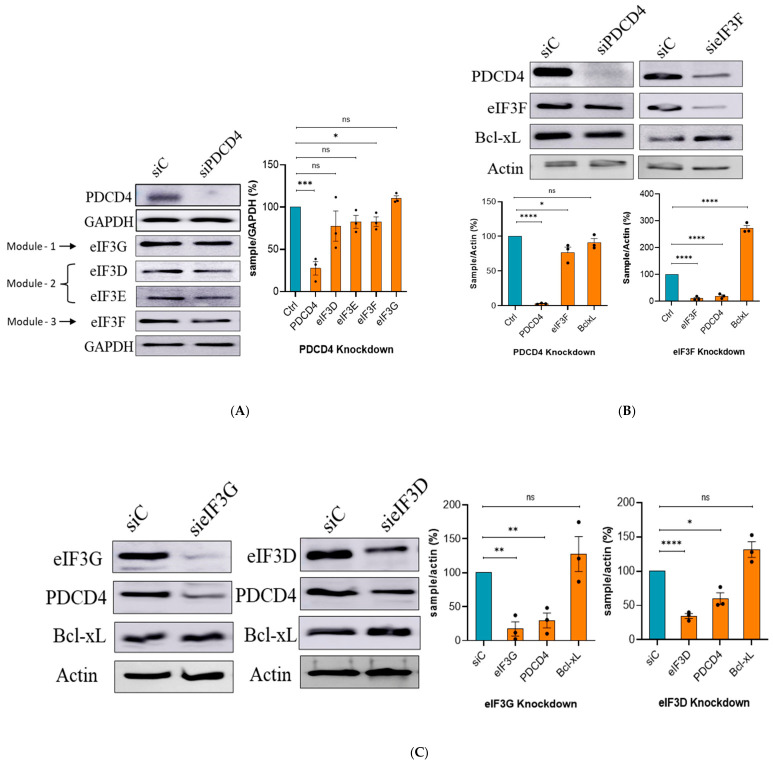
eIF3F depletion in U343 cells enhances the levels of Bcl-xL: (**A**) under PDCD4 depletion conditions, levels of eIF3D (module two), eIF3E (module two), eIF3F (module three), and eIF3G (module one) were reduced; (**B**) siRNA-mediated depletion of PDCD4 resulted in decreased levels of eIF3F, and a non-significant change in Bcl-xL.. When eIF3F was depleted, lower expression of PDCD4 was observed; however, the levels of Bcl-xL were robustly increased; (**C**) depletion of eIF3G decreased the levels of PDCD4 but Bcl-xL levels were not affected. Also, depletion of eIF3D modestly decreased the levels of PDCD4, and Bcl-xL levels were not significantly affected.

To investigate whether PDCD4 and eIF3 interactions impact their respective protein levels, PDCD4 was depleted using siRNA, and the different subunits of eIF3 were analyzed by Western blot. The levels of eIF3D, eIF3E, and eIF3G, were not robustly affected when PDCD4 was depleted in U343 cells (Figure 3A). However, the levels of eIF3F were modestly decreased under the PDCD4 depletion condition (Figure 3A and Figure S2A). Interestingly, when eIF3F was depleted using siRNA, the levels of PDCD4 were diminished in U343 cells (Figure 3B; right panel). Treatment of MG132 (a potent proteasomal inhibitor) prevented the degradation of PDCD4 in eIF3F-depleted U343 cells (Supplemental Figure S2A). Likewise, MG132 treatment also prevented the degradation of eIF3F in PDCD4-depleted U343 cells (Supplemental Figure S2A). These data suggest that the interaction of PDCD4 and eIF3F prevents their proteasomal degradation and that depletion of one leads to proteasomal degradation of the other. Based on these findings, we would like to suggest that the depletion of eIF3F would have activated proteasomal degradation of PDCD4.

It is known that eIF3 is a modular protein complex with three major modules [24]. Some evidence exists suggesting that depletion of one eIF3 subunit in either HeLa or HEK293 cells can lead to degradation of other subunits, and the eIF3 complex is disintegrated [24,25]. However, in U343 (GBM) cells, the depletion of eIF3G did not robustly affect the levels of eIF3F or eIF3D (Supplemental Figure S2B; top panel). The depletion of eIF3D did not affect the levels of eIF3F or eIF3G (Supplemental Figure S2B; top panel). Likewise, depletion of eIF3F did not change the levels of eIF3D or eIF3G (Supplemental Figure S2B; bottom panel). This can be attributed to the difference in the cell lines used in this study or GBM biology. We further tested one subunit from each module (eIF3G from module one, eIF3D from module two, and eIF3F from module three) for its role in Bcl-xL expression (Figure 3B,C). The depletion of eIF3F (a part of module three) in U343 cells resulted in a statistically significant increase in Bcl-xL levels (Figure 3B). However, the levels of Bcl-xL were not affected by depletion of eIF3G (a known direct interaction partner of PDCD4; a part of module one of eIF3) or eIF3D (a part of module two of eIF3) in U343 cells (Figure 3C). It is important to note that depletion of eIF3F, eIF3D, or eIF3G decreased the levels of PDCD4 protein. However, the levels of Bcl-xL protein were significantly enhanced only when eIF3F was depleted in U343 cells (Figure 3B,C). The global protein synthesis was not significantly affected by depletion of PDCD4 or eIF3F as revealed by the puromycin incorporation assay (Supplemental Figure S2C). These findings suggest an exclusive role of PDCD4–eIF3F in the expression of Bcl-xL. These findings also underscore the reciprocal regulatory influence between PDCD4 and eIF3F, suggesting their joint role in the regulation of Bcl-xL expression.

### 2.4. PDCD4 and eIF3F Work Together in Regulating Bcl-xL Expression

**Figure 4:**
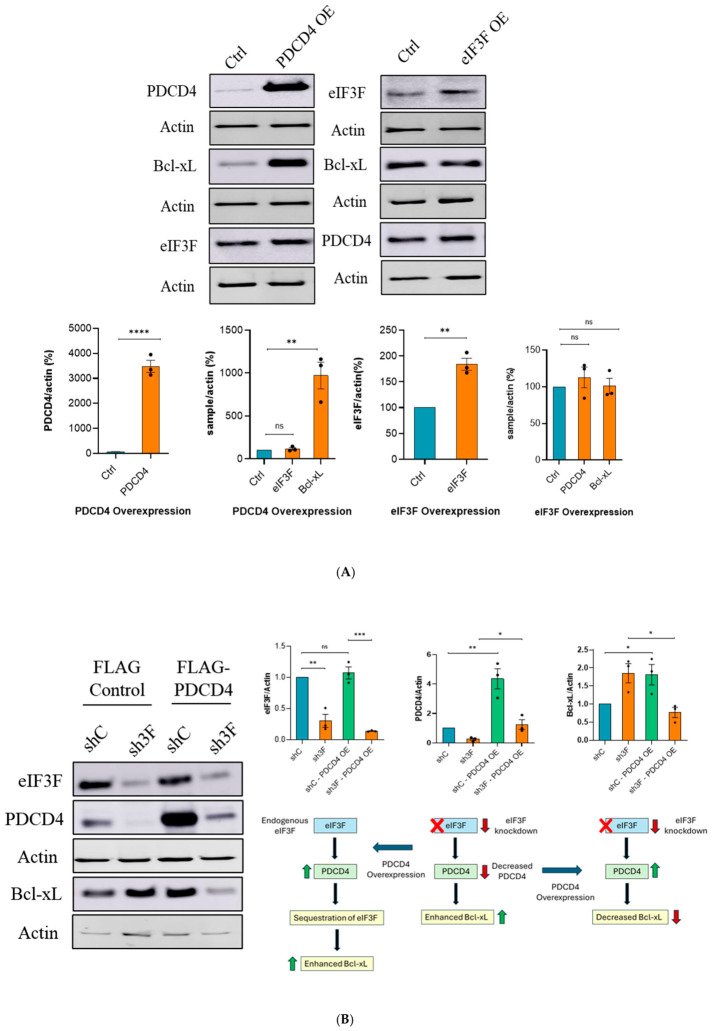
PDCD4 and eIF3F work together in regulating the levels of Bcl-xL: (**A**) overexpression of PDCD4 resulted in a robust enhancement of Bcl-xL levels without affecting the levels of eIF3F. eIF3F overexpression showed a significant (but not robust) decrease in Bcl-xL without affecting the levels of PDCD4; (**B**) we first stably depleted eIF3F using shRNA and subsequently overexpressed FLAG-PDCD4; (**B**; lane 1 vs. 2) as expected, shRNA-mediated depletion of eIF3F resulted in enhanced expression of Bcl-xL; (**B**; lane 1 vs. 3) overexpression of PDCD4 under stable expression of control shRNA also showed enhanced expression of Bcl-xL; (**B**; lane 3 vs. 4) however, overexpression of PDCD4 under the eIF3F depletion condition resulted in a modest but significant decrease in Bcl-xL levels. Green (up) arrows: upregulation/enhanced expression. Red (down) arrows: downregulation/decreased expression.

To elucidate the mechanism of Bcl-xL regulation by the PDCD4–eIF3F complex, we exogenously overexpressed PDCD4 or eIF3F in U343 cells. The overexpression of PDCD4 resulted in a significant enhancement of Bcl-xL levels. However, the levels of eIF3F were not affected by PDCD4 overexpression (Figure 4A). eIF3F overexpression did not affect the levels of PDCD4 and Bcl-xL (Figure 4A). To examine whether PDCD4 and eIF3F require each other to regulate Bcl-xL expression, we conducted experiments involving the overexpression of PDCD4 in eIF3F-depleted U343 cells. In this regard, we first stably depleted eIF3F using shRNA and subsequently overexpressed FLAG-PDCD4 (Figure 4B). As expected, shRNA-mediated depletion of eIF3F resulted in enhanced expression of Bcl-xL (Figure 4B; lane 1 vs. 2). Overexpression of PDCD4 under stable expression of control shRNA also showed enhanced expression of Bcl-xL (Figure 4B; lane 1 vs. 3). However, overexpression of PDCD4 under the eIF3F depletion condition resulted in a significant decrease in Bcl-xL levels (Figure 4B; lane 3 vs. 4). As illustrated in a model (Figure 4B; lower right panel), these findings suggest that eIF3F has an inhibitory role in Bcl-xL expression, and eIF3F depletion results in the derepression of Bcl-xL expression. PDCD4 overexpression, likely via sequestration of eIF3F, results in the derepression of Bcl-xL expression. Moreover, we can achieve only modest overexpression of PDCD4 in eIF3F-depleted cells (Figure 4B; lane 2 vs. 4). This could be likely due to the instability of PDCD4 under eIF3F depletion. Under eIF3F depletion + PDCD4 overexpression, Bcl-xL levels were decreased (Figure 4B; lane 2 vs. 4), suggesting that PDCD4 inhibits Bcl-xL expression when eIF3F is reduced. These data also suggest that both PDCD4 and eIF3F work together in regulating Bcl-xL expression. An overall summary of the findings that support the notion that eIF3F is required for PDCD4 activity can be demonstrated by a proposed model shown in Figure 4B (lower right panel). These data also suggest that both PDCD4 and eIF3F work together in regulating Bcl-xL expression.

### 2.5. PDCD4 and eIF3F Individually Interact with Bcl-xL mRNA

**Figure 5:**
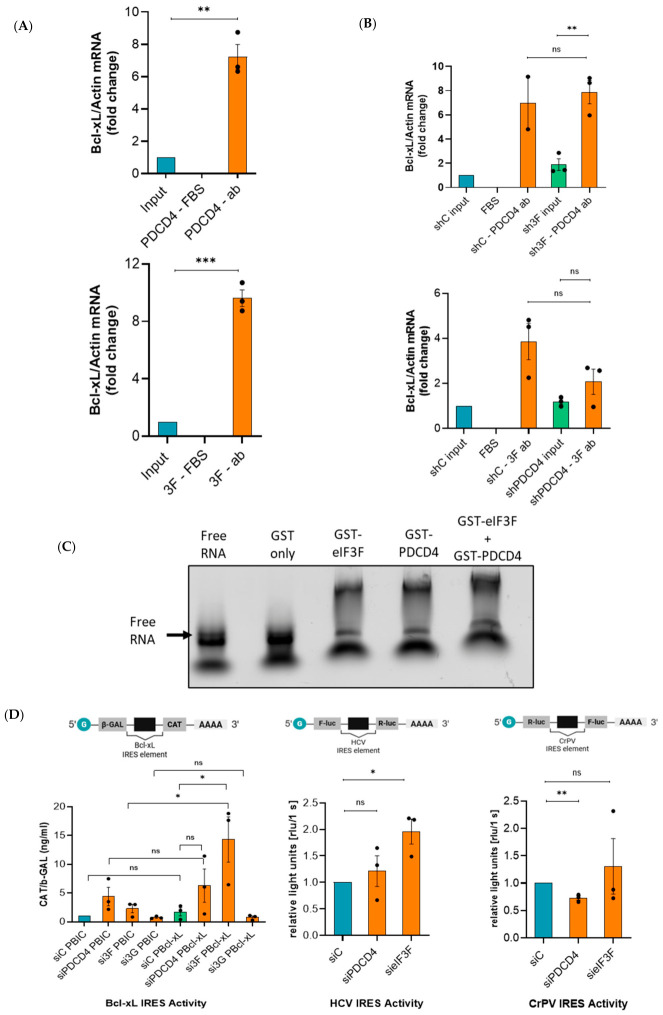
PDCD4 and eIF3F interact with the Bcl-xL mRNA and eIF3F depletion enhances Bcl-xL IRES activity: (**A**) Endo-RNA-IP results show that PDCD4 and eIF3F interact with Bcl-xL RNA. There was a significant increase observed between PDCD4 and eIF3F association with Bcl-xL RNA. FBS was used as a negative control, and all samples were normalized internally with actin mRNA. Input samples were used for the normalization of PDCD4-ab, and eIF3F-ab samples; (**B**) shRNA-mediated depletion of eIF3F did not enhance the interaction of PDCD4 with Bcl-xL mRNA; (**B**; **top** panel) also, shRNA-mediated depletion of PDCD4 did not enhance the interaction of eIF3F with Bcl-xL mRNA (**B**; **bottom** panel); (**C**) the direct interaction between GST-eIF3F (1.8 µM) and GST -PDCD4 (0.8 µM) with Bcl-xL IRES element of the 5′UTR shows a band retardation compared to free RNA and GST + RNA on the EMSA gel. The final concentration of RNA is 2 nM. The final lane represents the combination of GST-eIF3F and GST-PDCD4 at their respective concentrations; (**D**) bicistronic reporter (β-gal/CAT) assay showed that depletion of PDCD4 did not affect Bcl-xL IRES activity, whereas the depletion of eIF3F robustly and significantly enhanced Bcl-xL IRES activity. However, as expected, Bcl-xL IRES activity was not affected by the depletion of eIF3G (control); (**D**; **left** panel). A bicistronic Luciferase IRES assay was performed for HCV (Rluc/Fluc) and CrPV (Fluc/Rluc).

The observation of RNA-independent interaction between PDCD4 and eIF3F (Figure 1) raises the question of their individual associations with Bcl-xL RNA. To this end, we perform Endo-RNA-IP using PDCD4 or eIF3F antibodies. We performed RT-qPCR analysis to analyze the interaction of Bcl-xL mRNA with PDCD4 and eIF3F. Compared to the negative control (FBS), the levels of Bcl-xL mRNA were several-fold higher in PDCD4 and eIF3F Endo-IP (Figure 5A), suggesting that Bcl-xL mRNA was enriched in these Endo-IPs compared to input (lysate) (Figure 5A). This also suggests that both PDCD4 and eIF3F bind to the Bcl-xL RNA (Figure 5A). We then wanted to investigate if the levels of PDCD4 and eIF3F affect each other’s ability to interact with Bcl-xL mRNA. In this regard, we depleted PDCD4 or eIF3F from U343 cells using shRNAs. Subsequently, we performed Endo-RNA-IP as mentioned above. Under the PDCD4 depletion condition, eIF3F efficiently interacted with Bcl-xL mRNA (Figure 5B). Likewise, under the eIF3F depletion condition, PDCD4 efficiently interacted with Bcl-xL mRNA (Figure 5B). There was no significant difference in the interaction of Bcl-xL mRNA with PDCD4 or eIF3F under depletion of eIF3F or PDCD4, respectively. This suggests that PDCD4 and eIF3F neither enhance nor inhibit each other’s interaction with Bcl-xL mRNA. Both these protein factors bind independently to Bcl-xL mRNA, and they do not compete or cooperate to bind to Bcl-xL mRNA (Figure 5A,B). However, this data does not confirm if eIF3F or PDCD4 binds directly to Bcl-xL IRES RNA. Accordingly, we performed an in vitro electrophoretic mobility shift assay (EMSA) using fluorescently labelled Bcl-xL IRES RNA. We first purified recombinant GST-eIF3F, GST-PDCD4, and GST to homogeneity (Supplemental Figure S3A). Then, we optimized the concentrations of GST-eIF3F and GST-PDCD4 for the EMSA (Supplemental Figure S3B). We observed that 0.2–1.2 µM of GST-PDCD4 or 0.4–2.6 µM of GST-eIF3F was able to efficiently retard the mobility of Bcl-xL IRES RNA (Supplemental Figure S3B). Interestingly, we also noticed a retardation in mobility when a combination of GST-eIF3F (1.8 µM) and GST-PDCD4 (0.8 µM) was run with Bcl-xL IRES RNA (Figure 5C). We have observed that PDCD4 with a K_d_ value of 0.4 µM, has a slightly higher affinity to Bcl-xL IRES RNA compared to eIF3F with a K_d_ value of 0.8 µM. However, 1.3 µM purified GST did not show any effect on the mobility of Bcl-xL IRES RNA (Figure 5C and Figure S3B), suggesting that PDCD4 and eIF3F individually interact directly with Bcl-xL IRES RNA.

### 2.6. Depletion of eIF3F Enhances the Translation of Bcl-xL mRNA

As the 5′ UTR of Bcl-xL mRNA is known to harbour IRES element [26], we investigated the ability of PDCD4 and eIF3F to regulate IRES-mediated translation of Bcl-xL in U343 cells. We depleted eIF3G (negative control), eIF3F, or PDCD4 from U343 cells. Subsequently, cells were transfected with plasmids containing the previously described bicistronic reporters, β-galactosidase (β-gal), and chloramphenicol acetyltransferase (CAT), including Bcl-xL IRES element in between them (pBIC as control and pBIC-Bcl-xL IRES including the IRES element) [17]. The IRES activity was measured by taking a ratio of CAT to β-gal levels (Figure 5D; right top panel and Supplemental Table S4). As expected, Bcl-xL IRES activity was not affected by eIF3G depletion (Figure 5D; left panel). Also, there was a non-significant change in IRES activity when PDCD4 was depleted. However, depletion of eIF3F from U343 cells resulted in a significantly increased level of Bcl-xL IRES activity (Figure 5D; left panel). As CrPV intergenic region (IGR) IRES mimics the initiator tRNA structure and does not require eIF3 for the ribosome recruitment [27], we used CrPV IGR IRES as a negative control. The IRES activity was measured by taking a ratio of FLuc to RLuc levels (Figure 5D; right panel and Supplemental Table S5). CrPV IRES activity was not affected by the depletion of eIF3F (Figure 5D, right panel). However, CrPV IRES activity was modestly but significantly decreased by the depletion of PDCD4 (Figure 5D, right panel), suggesting that CrPV IRES activity can be modestly affected by PDCD4. Moreover, several papers demonstrated that HCV IRES RNA interacts with the eIF3 complex [21,28]. Therefore, we measured HCV IRES activity in eIF3F- or PDCD4-depleted U343 cells. The IRES activity was measured by taking a ratio of RLuc to FLuc levels (Figure 5D; middle panel and Supplemental Table S5). To our surprise, like Bcl-xL IRES, PDCD4 depletion did not significantly affect HCV IRES activity, and eIF3F depletion significantly enhanced HCV IRES activity (Figure 5D; middle panel). This suggests that eIF3F normally inhibits IRES-mediated translation of both Bcl-xL, and HCV RNA.

**Figure 6:**
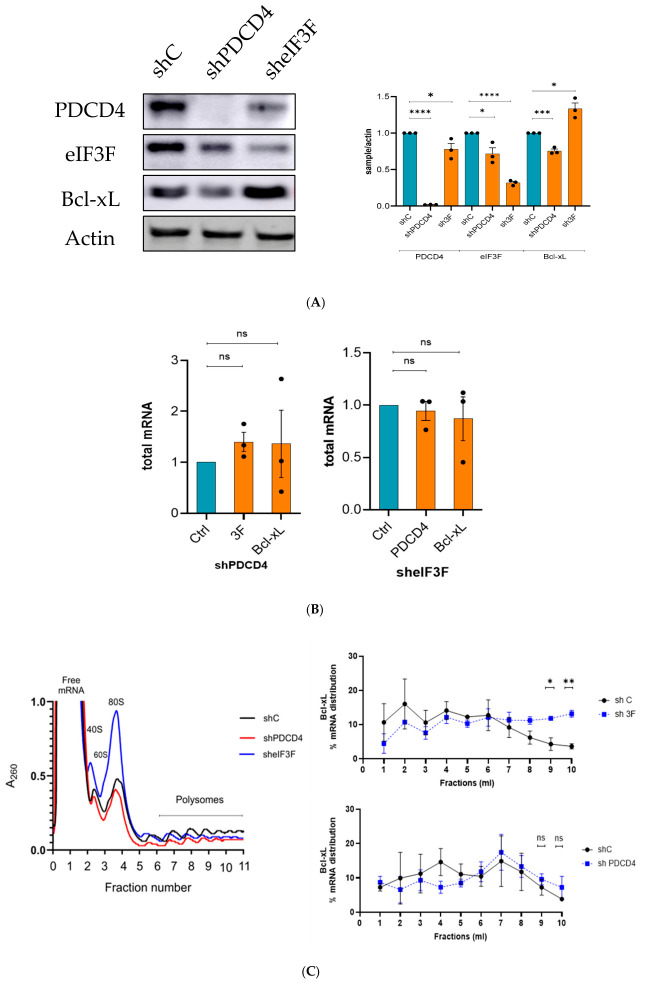
eIF3F depletion enhanced the translation of Bcl-xL mRNA: (**A**) shRNA-mediated depletion of PDCD4 and eIF3F was confirmed by Western blot analysis. The levels of Bcl-xL were also observed. eIF3F depletion showed a significant increase in Bcl-xL; (**B**) total mRNA levels of eIF3F and Bcl-xL were analyzed in cells transduced with shPDCD4. Total mRNA levels of PDCD4 and Bcl-xL were analyzed in cells transduced with sheIF3F; (**C**; **left** panel) polysome profile of shC, shPDCD4, and sheIF3F showing the monosomes and polysomes levels across multiple fractions; (**C**; **right** panel) percentage RNA abundance across polysome fractions. Polysome profiling fractions 9–10 show that Bcl-xL mRNA translation was significantly increased in eIF3F depletion condition. Depletion of PDCD4 did not significantly affect the mRNA translation of Bcl-xL.

To verify that the translation of endogenous Bcl-xL mRNA is regulated by eIF3F or PDCD4, we performed a polysome profiling assay. To this end, we efficiently depleted PDCD4 or eIF3F from U343 cells using shRNAs (Figure 6A). shRNA-mediated depletion of PDCD4 did not significantly affect the steady-state mRNA levels of eIF3F and Bcl-xL mRNAs (Figure 6B; left panel). Likewise, shRNA-mediated depletion of eIF3F did not significantly affect the steady-state mRNA levels of PDCD4 and Bcl-xL (Figure 6B; right panel). We separated monosomes and polysomes in a sucrose density gradient using ultracentrifugation from the control, PDCD4- or eIF3F-depleted U343 cells (Figure 6C; left panel). The collected fractions were subjected to RT-qPCR analysis using ΔΔCt method relative to actin mRNA levels to determine the % distribution of Bcl-xL (Figure 6C; right panel), eIF3F, or PDCD4 (Supplemental Figure S4) mRNA across 10 fractions of the polysome profile. In PDCD4-depleted U343 cells, the translation of mRNAs encoding eIF3F and Bcl-xL did not change significantly (Figure 6C; right panel and Supplemental Figure S4). Additionally, under eIF3F-depletion conditions, the translation of PDCD4 mRNA did not change (Supplemental Figure S4). However, the translation of Bcl-xL mRNA was significantly increased in eIF3F-depleted U343 cells (Figure 6C; right panel).

### 2.7. eIF3F–PDCD4-Dependent Regulation of Bcl-xL Is Observed in the Tested GBM Lines

**Figure 7:**
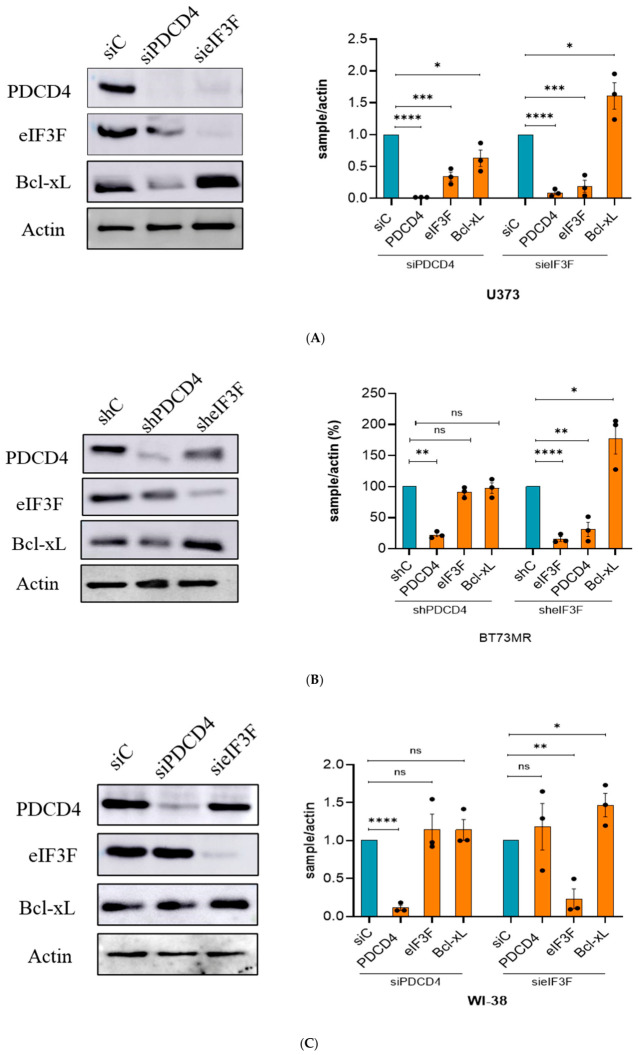
The eIF3F–PDCD4-dependent mechanism is implicated in glioblastoma cells: (**A**) eIF3F depletion in U373 cells robustly enhanced the levels of Bcl-xL; also, the depletion of PDCD4 in U373 cells resulted in a significant decrease in Bcl-xL levels; depletion of eIF3F, and PDCD4 decreased the levels of PDCD4 and eIF3F, respectively; (**B**) eIF3F depletion in BT73MR robustly enhanced the levels of Bcl-xL. Also, the depletion of PDCD4 resulted in a no significant change in Bcl-xL levels; depletion of eIF3F, and PDCD4 decreased the levels of PDCD4 and eIF3F, respectively; (**C**) WI-38 did not show decreased levels of eIF3F in PDCD4 depletion or vice versa, which is strikingly different from GBM cells; The levels of Bcl-xL in eIF3F depletion were significantly high; however, not as robust compared to GBM cells; WI-38 cells did not show decreased levels of eIF3F in PDCD4 depletion or vice versa.

Since we did not see the S6K-eIF3-PDCD4 axis functioning in MEFs, we wondered if this is unique to cancer cells. Accordingly, we first compared the levels of PDCD4, eIF3F, and Bcl-xL in GBM cells (U343 and BT73MR) with those in WI-38 (human lung fibroblasts) cells (Supplemental Figure S5). Because eIF3F and PDCD4 are tumour suppressor proteins, their levels were low in GBM cells compared to WI-38. As expected, the levels of Bcl-xL were low in WI-38 cells compared to GBM cells (Supplemental Figure S5). Subsequently, to check if PDCD4 and eIF3F regulate Bcl-xL differently in GBM vs. non-cancer fibroblasts, we depleted PDCD4 or eIF3F in three different glioblastoma cell lines, one of which is a primary brain tumour stem cell (BTSC) line. For comparison, we depleted PDCD4 or eIF3F in human lung fibroblasts. Subsequently, we performed Western blot analysis for PDCD4, eIF3F, and Bcl-xL in all four cell lines. The depletion of PDCD4 resulted in a modest decrease in Bcl-xL levels in all these experiments (Figure 3B, Figure 6A and Figure 7). However, in the case of siRNA-mediated depletion of PDCD4 in U373 (Figure 7A) and shRNA-mediated depletion of PDCD4 in U343 (Figure 6A), we observed a modest but statistically significant decrease in Bcl-xL levels. However, in all these cases, the effect of PDCD4 depletion on Bcl-xL levels was not robust. We observed significantly increased levels of Bcl-xL under eIF3F depletion conditions in U343 (Figure 3B), U373 (Figure 7A), and BT73MR (Figure 7B) cells. In contrast, when PDCD4 was depleted in human lung fibroblast (WI-38) cells, the levels of eIF3F and Bcl-xL were not affected (Figure 7C). However, there was a modest increase in PDCD4 and Bcl-xL levels in eIF3F-depleted WI-38 cells (Figure 7C). These results suggest that the PDCD4–eIF3F-mediated regulation of Bcl-xL mRNA translation is likely more relevant for the tested glioblastoma cell lines.

## 3. Discussion

IRES-mediated translation initiation is one of the key modes of non-canonical translation initiation that regulates the translation of a subset of mRNA [1,2,29]. Largely, the translation of stress-related mRNAs (such as pro- and anti-apoptotic proteins) is regulated by IRES-mediated translation initiation [30,31]. IRES transacting factors (ITAFs) play an important role in regulating IRES-mediated translation initiation [2]. Investigating the role of these ITAFs in IRES-mediated translation initiation is critical, as they can direct the translation of stress-related mRNAs under cellular stress conditions [2]. PDCD4 is a well-known tumour suppressor protein, which binds to eIF4A and PABP to regulate canonical translation initiation [13]. Our previous work established PDCD4 as an ITAF that regulates IRES-mediated translation of XIAP and Bcl-xL mRNAs [12,17]. eIF3 is a multi-subunit complex that is shown to regulate both global and transcript-specific translation [29,32]. For example, hepatitis C virus (HCV) IRES forms a complex with eIF3 and facilitates the translation of HCV mRNA [33]. Also, reports exist suggesting that eIF3 may have an inhibitory role in HCV IRES-mediated translation [34]. Previously, we have shown that eIF3 binds to XIAP IRES RNA in an RNA secondary structure-dependent manner and facilitates the formation of the translation initiation complex on XIAP IRES RNA [22].

Both PDCD4 and eIF3 are targets of the mTOR pathway [35], and are phosphorylated by S6K1 and/or 2, a downstream target of mTOR [35]. PDCD4 phosphorylation promotes PDCD4 degradation via ubiquitination while eIF3 phosphorylation enhances eIF3 activity [20,35]. Specifically, S6K directly interacts with eIF3F and phosphorylates eIF3G that enhances mRNA translation [20]. One of the known common target mRNAs for PDCD4 and eIF3 is XIAP mRNA [17,32]. PDCD4 and eIF3 directly interact with XIAP IRES RNA and have opposing effects on XIAP mRNA translation initiation [17,32]. PDCD4 is also shown to directly interact with Bcl-xL IRES RNA and to inhibit IRES-mediated translation of Bcl-xL [17]. A recent publication has shown that the PDCD4-N-terminus domain (NTD) directly interacts with eIF3G by forming an intermolecular β-sheet when PDCD4 occupies the mRNA channel on the 40S ribosome. Also, eIF3I forms a weak interaction with the C-terminus domain (CTD) of PDCD4 and interferes with PDCD4’s interaction with eIF4A [16]. Moreover, PDCD4 was shown to interact with eIF3H and to enhance the metastasis of lung adenocarcinoma [36]. However, the role of PDCD4–eIF3 (or its subunits) interaction in the regulation of mRNA translation remained largely unexplored. Hence, this study investigates the interaction between PDCD4 and eIF3 and its impact on the IRES-mediated translation of Bcl-xL mRNA.

eIF3 is a large 12-subunit modular complex (recent evidence points that eIF3J is not considered a subunit) [37,38] of which 10 subunits, spanning all three modules, (eIF3A, eIF3B, eIF3C, eIF3D, eIF3E, eIF3F, eIF3G, eIF3I, eIF3L, and eIF3M) interact with PDCD4 in the IP-MS experiment (Figure 1A). Interaction of at least three of these subunits (eIF3D, eID3F and, eIF3G) with PDCD4 was further confirmed by IP experiment followed Western blot analysis (Figure 1B). Moreover, the depletion of PDCD4 results in the proteasomal degradation of eIF3F (Supplemental Figure S2A). Not only do PDCD4 and eIF3F interact with each other, but we also show that they regulate each other at the protein level. When PDCD4 was depleted, we observed a modest decrease in eIF3F (Figure 3A and Figure S2A). When we depleted eIF3F, eIF3G, or eIF3D, we observed a reduction in PDCD4 levels (Figure 3B,C). These observations suggest that eIF3 has a stabilizing effect on PDCD4. It is possible that eIF3 subunit(s) mask the S6K-dependent phosphorylation sites of PDCD4 and, as a result of eIF3 subunit(s) depletion, PDCD4 is destabilized via the proteasomal degradation.

PDCD4 might establish direct interactions with individual subunits such as eIF3G, eIF3I, and eIF3H [16,36], and as demonstrated for eIF3F in this study (Figure 1E). It is possible that PDCD4 could interact with a subcomplex(s) of eIF3 subunits, leading to the inhibition of translation. PDCD4’s interaction with one subunit or a subcomplex of eIF3 could prevent the formation of a functional eIF3 complex or prevent eIF3’s autonomous activity. Similarly, PDCD4 binding to eIF3B and eIF3E may also affect interaction with eIF4G and inhibit ribosome recruitment to the mRNA. PDCD4’s interaction with eIF3D could hinder eIF3D’s cap binding activity and could inhibit specialized translation [39]. These canonical and specialized functions of the eIF3 subunits can be affected by their interaction with PDCD4. Besides eIF3, PDCD4 is known to interact with PABP and eIF4A to suppress their role in translation initiation [13,14]. Therefore, the interaction between PDCD4 and eIF3 has the potential to impede eIF3’s association with mRNA, ribosome, or other cellular proteins, thereby exerting inhibitory effects on translation.

As both PDCD4 and eIF3F directly bind to S6K [17,20], we hypothesized a coregulation of PDCD4-S6K-eIF3F complex, and its critical role in the IRES-mediated translation of Bcl-xL. To this end, we systematically investigated the interaction between PDCD4 and eIF3F and demonstrated that PDCD4 and eIF3F interact directly with each other (Figure 1A–F). eIF3F has also been reported earlier to interact with S6K1 and was demonstrated by an in vitro experiment [20]. S6K1 binds eIF3F to phosphorylate eIF3G subunit [20]. However, the in cellulo experiments suggested that both S6K1 and 2 have a role in eIF3 phosphorylation [20]. PDCD4 interacting with eIF3F can interfere with eIF3F and S6K1/2 association to prevent phosphorylation of eIF3G and overall inhibition of translation. Therefore, we hypothesized that S6K1/2 would modulate the interaction between eIF3F and PDCD4. In this regard, we performed an IP experiment using serum-starved cells and showed that PDCD4–eIF3F interaction was not affected (Figure 2B). Treatment with serum-free media could trigger multiple effects besides S6K1 and 2 inhibitions. Therefore, it is important to perform similar co-IP experiments using chemical inhibitors that specifically target S6K1 and 2 activities. Pharmacological inhibition of the mTOR/S6K1/2 axis did not affect the PDCD4–eIF3F interaction (Figure 2D and Figure S1C). Moreover, the IP experiment performed from S6K double knockout MEFs also confirmed that the S6K1/2 axis does not affect PDCD4–eIF3F interaction. Thereby, we disproved our hypothesis of S6K regulating the interaction of PDCD4 and eIF3F.

As depletion of eIF3F or PDCD4 affects each other’s protein level, one can expect that Bcl-xL levels would increase in both eIF3F- and PDCD4-depleted cells. However, it is important to note that the depletion of eIF3F results in an extremely low level of PDCD4. In contrast, under PDCD4 depletion conditions, the decrease in eIF3F level was modest (Figure 3). When PDCD4 was overexpressed in U343 cells, the levels of Bcl-xL were significantly increased (Figure 4A). This could be explained by the possibility that eIF3F gets sequestered by PDCD4 overexpression resulting in the increased expression of Bcl-xL. When PDCD4 was over-expressed in eIF3F-depleted conditions, due to the absence of endogenous eIF3F, PDCD4 exerted a modest inhibitory effect on Bcl-xL expression (Figure 4B). Therefore, it is very likely that PDCD4 and eIF3F work in synergy to regulate the IRES-mediated translation of anti-apoptotic protein Bcl-xL.

We further analyzed the effect of PDCD4 and eIF3F on IRES-mediated translation initiation of Bcl-xL. The results of the bicistronic reporter assay indicated that the levels of IRES activity were higher in the eIF3F knockdown condition compared to PDCD4 depletion (Figure 5D). Depletion of eIF3G (part of module one of eIF3), a known interaction partner of PDCD4 [16], did not enhance Bcl-xL IRES activity (Figure 5D). As HCV IRES is known to form a complex with eIF3 [21,28], we measured the HCV IRES activity in PDCD4- or eIF3F-depleted U343 cells. While PDCD4 depletion did not affect HCV IRES activity, like Bcl-xL IRES, the activity of HCV IRES was significantly increased when eIF3F was depleted (Figure 5D). Of note, cryptic promoter activity and spurious splicing activities have been observed in some constructs of cellular IRESes [40], which were not ruled out for the Bcl-xL IRES assay (Figure 5D). However, the translational regulation of Bcl-xL was further confirmed by performing a polysome profiling experiment (Figure 6). eIF3F is a tumour suppressor that is downregulated in many cancers [32] and its role in translation inhibition is known [41,42]. The inhibitory role of eIF3F in transcript-specific translation has been reviewed by Dieter A. Wolf [43]. Therefore, it is not surprising that eIF3F depletion enhanced the IRES activity of both Bcl-xL and HCV. However, the activity of CrPV IRES, which mimics the initiator tRNA structure, was not increased in eIF3F-depleted cells (Figure 5D). We have previously shown that CrPV IRES does not bind to eIF3 [22]. Therefore, it was expected that CrPV IRES activity would not be significantly affected by eIF3F depletion. Surprisingly, CrPV IRES activity was modestly decreased in PDCD4-depleted cells. PDCD4 may act as an ITAF for CrPV IRES, which we have not confirmed in this study. By performing a polysome profiling experiment, we have demonstrated that eIF3F regulates Bcl-xL expression at the mRNA translation level. There is a robust increase in Bcl-xL mRNA translation when eIF3F is depleted; however, there was no statistically significant difference in Bcl-xL mRNA translation when PDCD4 was depleted (Figure 6). This finding does not corroborate our previous study completely, in which we showed that depletion of PDCD4 in HEK293T cells enhanced the translation of Bcl-xL mRNA [17]. However, this study was not performed in the context of eIF3 (or eIF3F). As mentioned above, overexpression of PDCD4 decreased Bcl-xL expression when eIF3F was depleted (Figure 4B). Here, we have shown that PDCD4 and eIF3F together play a critical role in Bcl-xL mRNA translation in GBM cells. Exogenous overexpression of PDCD4 led to enhanced expression of Bcl-xL protein (Figure 4). As such, PDCD4 overexpression has a similar effect as eIF3F depletion on Bcl-xL protein levels. Therefore, we suggest that overexpression of PDCD4 would likely sequester eIF3F, leading to enhanced expression of Bcl-xL. Overall, these experiments highlight that PDCD4 and eIF3F collaborate with each other and form a complex to regulate the translation of Bcl-xL.

Puromycin incorporation assay reported that there was no increase in overall protein synthesis in PDCD4 and eIF3F depletion (Supplemental Figure S2A). Hence, the elevation in Bcl-xL protein levels can be attributed to the enhanced IRES-mediated translation of Bcl-xL mRNA. In eIF3F depletion, we did not detect a decrease in PDCD4 mRNA translation (Supplemental Figure S4). Thus, the decrease in PDCD4 protein levels under eIF3F depletion could likely be explained by the regulation of PDCD4 at the proteasomal level (Supplemental Figure S2A). Together, all the results suggest that PDCD4 and eIF3F interaction is crucial for the regulation of IRES-mediated translation of Bcl-xL mRNA. Furthermore, it is possible that PDCD4 exerts a negative regulatory influence on Bcl-xL expression by interacting with eIF3F. The overall study aims at deciphering the mechanism of the regulation of mRNA translation of an anti-apoptotic protein, Bcl-xL, by PDCD4 and eIF3. In summary, we have demonstrated that PDCD4 interacts with 10 eIF3 subunits and regulates the stability of some eIF3 subunits. PDCD4 interacts directly with eIF3F in an RNA-independent manner. Both PDCD4 and eIF3F, individually, directly interact with Bcl-xL IRES RNA. However, they do not facilitate or inhibit each other’s interaction with Bcl-xL RNA. Depletion of eIF3F destabilizes PDCD4 and enhances IRES-mediated translation of Bcl-xL mRNA. On the other hand, depletion of PDCD4 destabilizes the eIF3 complex, including eIF3F, but does not significantly affect the IRES-mediated translation of Bcl-xL mRNA. We have also shown that PDCD4 and eIF3F work together in regulating Bcl-xL translation and the mTOR/S6K1&2 axis does not regulate the interaction between eIF3F and PDCD4. Using the RNAi approach, we further functionally validated eIF3G (module one of eIF3), eIF3D (module two of eIF3), and eIF3F (module three of eIF3), one key subunit from each module, for their role in Bcl-xL expression. Bcl-xL levels were significantly enhanced only under eIF3F depletion conditions, but not when eIF3G or eIF3D was depleted. While we cannot be absolutely sure, at this time, that the remaining nine subunits are not taking part in Bcl-xL regulation, we have presented herein a solid set of data strongly suggesting that PDCD4–eIF3F is involved in the regulation of Bcl-xL translation. Although several publications suggest that the eIF3 complex plays an important role in IRES-mediated translation, the knowledge about the role of individual eIF3 subunits in global and transcript-specific translation is still emerging. Moreover, there is clear evidence suggesting that PDCD4 interacts with a subset (or individual) of eIF3 subunits [16,36] and plays an important role in translation regulation. Therefore, it seems that the interaction of PDCD4 with specific eIF3 subunits is mechanistically context-dependent. Moreover, by depleting tumour suppressors PDCD4 or eIF3F in at least three glioblastoma cell lines (including brain tumour stem cells) and non-cancer human lung fibroblasts, we show that PDCD4–eIF3F-dependent regulation of Bcl-xL is likely specific to the tested glioblastoma cells. Thereby, this mechanism may play an important role in glioblastoma pathophysiology. Understanding the PDCD4–eIF3-dependent mechanism of non-canonical translation of an antiapoptotic protein, Bcl-xL, has potential applications in developing anti-cancer therapeutics against ‘hard-to-treat’ and ‘high-fatality’ cancers such as glioblastoma.

## 4. Materials and Methods

### 4.1. Cell Lines and Constructs

Human glioblastoma cell line U343, U373, BT73MR (BTSCs), as well as HEK293T cells, WI-38, S6K double knockout mouse embryonic fibroblast cells (MEFs), and wild-type MEFs were used for the cellular assays. All mammalian cell lines (except BTSCs) were maintained in Dulbecco’s Modified Eagle’s Medium (DMEM) (Cytiva, Burnaby, BC, Canada) supplemented with 10% *v*/*v* fetal bovine serum (FBS), L-glutamine, and penicillin/streptomycin. BTSCs (BT73MR) were cultured in serum-free media supplemented with epidermal growth factor, fibroblast growth factor, and heparan sulphate in a humidified incubator (37°C, 5% CO_2_) as described elsewhere [44,45]. Both MEFs lines were obtained from Dr. Nahum Sonenberg, Department of Biochemistry, McGill University, Canada. The plasmids used to express proteins, and reporter constructs to monitor cap-dependent and cap-independent translation are tabulated as shown in Supplemental Table S1.

### 4.2. Plasmid Purification

A single colony of *E. coli* containing the above-mentioned plasmids from the LB agar was inoculated into a 3 mL LB broth containing 100 μg/mL final concentration of ampicillin. The 3 mL starter culture was incubated in a 37 °C shaker for 10 h. 1 mL from the starter culture was then inoculated into 200 mL LB broth containing 100 μg/mL final concentration of ampicillin. The 200 mL culture was then incubated overnight at 37 °C with constant shaking at 150 rpm. Following instructions, the plasmid was extracted from the following culture according to the Qiagen plasmid maxiprep kit. The quality of the plasmid purified was analyzed on a 0.8% *w*/*v* agarose gel and quantified using the Bio-Drop spectrophotometer (Montreal Biotech Inc., Montreal, QC, Canada).

### 4.3. Transfection

Plasmid transfections: Before transfection, cells were trypsinized and counted using the trypan blue staining. 3 × 10^6^ cells were seeded into a 100 mm tissue culture plate. Forty-eight hours after seeding, cells were transiently transfected with the pcDNA3-PDCD4-FLAG and pcDNA3-FLAG plasmid using Lipofectamine^TM^ 2000 transfection reagent (Thermo Fischer Scientific, Nepean, ON, Canada). The DNA plasmid, transfection reagent, and serum-free opti-MEM media were mixed in proportions per the manufacturer’s protocol and incubated at ambient temperature for 20 min. The transfection mix was then added to the 100 mm tissue culture dish containing a monolayer of mammalian cells. The cells were incubated at 37 °C for 48 h in a CO_2_ incubator before lysis. A total of 3 × 10^5^ cells were seeded into a 6-well tissue culture plate (following reverse transfections of siRNA, see below). Forty-eight hours after seeding, cells were transiently transfected with pcDNA3-FLAG, pcDNA3-FLAG-PDCD4, HA-eIF3F, βGAL-CAT (pBIC) and βGAL—Bcl-xL—IRES—CAT (pBcl-xL) plasmids (Supplemental Table S1) using Lipofectamine^TM^ 2000 transfection reagent (Thermo Fischer Scientific). The DNA plasmid, transfection reagent, and serum-free opti-MEM media were mixed in proportions per the manufacturer’s protocol and incubated at ambient temperature for 20 min. During the incubation period, cells in the 6-well plate were washed with opti-MEM (Thermo Fischer Scientific), and 750 μL of opti-MEM was added to each well. A total of 250 µL transfection mix was added to the respective wells. The cells were incubated with the transfection mix for 4–6 h, and then the opti-MEM media was replaced with DMEM containing 10% *v*/*v* FBS. The cells were incubated for 24 h and harvested.

siRNA Reverse transfections: In the case of siRNA transfection, the cells from a 100 mm tissue culture plate were treated with trypsin for 5 min at 37 °C. 3 × 10^5^ cells/well were seeded in a 6-well plate. Transfection mix containing Lipofectamine^®^ RNAiMAX (Thermo Fischer Scientific) transfection reagent, siRNA (siPDCD4, sieIF3D, sieIF3E, sieIF3F, sieIF3G—Horizon Discovery, Lafayette, CO, USA), and opti-MEM media (Thermo Fischer Scientific) were mixed according to the manufacturer’s protocol and incubated for 20 min at ambient temperature. 250 µL transfection mix was added to the respective wells. The plate was set at 37 °C for 96 h in a CO_2_ incubator and harvested.

### 4.4. shRNA-Mediated Depletion of PDCD4 and eIF3F

Lentiviral particles coding small hairpin (shRNA) specific for PDCD4, eIF3F, scrambled control was produced by transfecting 3.3 × 10^7^ HEK293T cells in a 100 mm tissue culture plate with 5.4 µg packaging plasmid (psPAX2), 0.6 µg envelope plasmid (VSVG), and 6 µg PDCD4/eIF3F shRNA plasmid (Supplemental Table S1). The transfection was carried out using the XTREME gene transfection reagent (Millipore Sigma, Oakville, ON, Canada) and Opti-MEM media (Thermo Fischer Scientific, Nepean, ON, Canada) for 18 h. Following this, the media was aspirated and replaced with high-Bovine Serum Albumin (BSA, Millipore Sigma) growth media (DMEM containing BSA). The media was harvested twice at 24 h and 48 h and pooled. The media containing lentiviruses were centrifuged at 1300 rpm for 2 min at ambient temperature to remove any cell debris. The supernatant was collected, passed through a 0.45 µm filter, aliquoted, and stored at −80 °C. The aliquot was thawed at room temperature prior to transduction. 1.5 × 10^6^ U343 cells were transduced in a 10 cm tissue culture plate with 1 mL lentivirus added dropwise and 5 µg polybrene. When the cells reached 70% confluency, the media was replaced with puromycin (Millipore Sigma) containing media for selection. At 96 h after transduction, the cells were washed with PBS and harvested for Western blot, and polysome profiling experiments. BTSCs (BT73MR) were dissociated with Accutase (Innovative Cell Technologies, San Diego, CA, USA), and the single cell suspension was obtained. The cells were seeded 250,000 cells/well in a 6-well plate. These cells were transduced with shC, shPDCD4, and sheIF3F lentivirus, respectively, as described elsewhere [46]. The cells were incubated for 24 h after which the media was changed and supplemented with puromycin (2 μg/mL) for antibiotic selection. The cells were then harvested after 48 h and lysates were used for Western blot analysis.

### 4.5. Drug/Inhibitor Treatments

U343 cells were reverse-transfected with siRNA as mentioned in the section above. In the case of siRNA transfections, the time was optimized to 96 h before harvesting. The cells were treated with optimized concentrations of 80 nM of AZD2014, and MG132 (Millipore Sigma) (1.8 mM) at 24 h and 16 h before harvesting.

### 4.6. Immunoprecipitation (IP)

FLAG-IP: U343 cells were transfected with pcDNA3-FLAG or pcDNA3-PDCD4-FLAG plasmids. Post 48 h of transfection, the cells were treated with 3.4% *v*/*v* formaldehyde for 10 min, followed by 10 min of incubation with 0.02 mM glycine to remove the excess formaldehyde. The cells were washed with PBS, and then the cells were lysed in 1.1 mL of RIPA buffer. The lysate was centrifuged at 10,000× *g* for 10 min. Exactly 1 mL of the lysate was added to 40 μL of equilibrated ANTI-FLAG^®^ M2 Affinity Gel (Millipore Sigma). The lysate was incubated with the affinity gel in the cyclomixer for 4 h at 4 °C. After incubation, the beads were isolated by centrifuging at 5000× *g* for 30 s. The matrix was washed with 500 μL of 1X PBS four times. The proteins were eluted by adding 40 μL of 2X concentration Laemmli sample buffer (Bio-Rad, Mississauga, ON, Canada) and heating for 10 min at 98 °C. The samples were analyzed using the Western blotting technique. Exactly 1% of the total cell lysate used for IP was loaded in the SDS-PAGE and co-IP samples as an input control.

eIF3F IP: 40 μL of protein G Dynabeads™ (Invitrogen, Burlington, ON, Canada) was incubated with 1 μg of eIF3F or PAIP1 (negative control) specific antibody (Supplemental Table S2) for 20 min at room temperature, followed by 1 h at 4°C. Unbound antibody was washed with PBS and incubated with cell lysate prepared for FLAG-immunoprecipitation. The lysate and beads were incubated for 4 h at 4 °C. Post incubation, the beads were separated using a magnetic rack and washed with 500 μL of 1XPBS. Proteins bound to beads were eluted with 40 μL of 2X Laemmli sample buffer and analyzed by Western blotting. Exactly 1% of the total lysate volume used for IP was used as an input control during Western blot analysis.

FLAG-IP to detect RNA-independent protein–protein interaction: Two sets of 100 mm tissue culture dishes containing U343 cells were transfected with the pcDNA3-FLAG or pcDNA3-PDCD4-FLAG plasmids. After 48 h of transfection, set one was harvested in 1 mL of the RIPA buffer supplemented with 250 μg of RNaseA, and set two was harvested in 1 mL of the RIPA buffer supplemented with 200 units of RNase inhibitor. The lysate was centrifuged at 10,000× *g* for 10 min at 4 °C to separate the cell debris. Then the supernatant was used to perform FLAG-IP similarly as mentioned above.

Endogenous IP (Endo-IP): A total of 100 μL of protein G Dynabeads™ (Invitrogen) was incubated with PDCD4 specific antibody (Supplemental Table S2) (1:25 in 0.02% 1×X PBST) or FBS (negative control) for 20 min at room temperature, followed by 4 h at 4°C. Then, 100 mm culture plates were seeded with U343 and when 80% confluency was reached, they were treated with 10 µL of DMSO or 80 nM of AZD2014. The plates were washed twice with cold 1X PBS and collected using 1 ml RIPA buffer with protease and phosphatase inhibitors. The antibody-coated beads were washed with PBS and incubated with U343 cell lysates. The lysate and beads were incubated overnight at 4 °C. The beads were separated using a magnetic rack and washed with 500 μL of cold 1×X PBS three times. Proteins bound to beads were treated with 40 μL of 2×X Laemmli sample buffer at 95 °C for 10 min and analyzed by Western blotting. Exactly 3% of the total lysate volume used for IP was used as an input control during Western blot analysis. To test the effect of mTOR inhibition on PDCD4 and eIF3F interaction, Endo-IP was performed as mentioned above from U343 cells treated with DMSO or 80 nM of AZD2014.

Endogenous RNA IP (Endo-RNA-IP): A total of 100 μL of protein G Dynabeads™ (Invitrogen) was incubated with PDCD4 and eIF3F specific antibody (1:25 in 0.02% 1×X PBST) or FBS (negative control) for 20 min at room temperature, followed by 4 h at 4 °C. Then, 100 mm culture plates were seeded with U343 and when 80% confluency was reached, the plates were washed twice with cold 1×X PBS and collected using 1 mL RIPA buffer with protease and phosphatase inhibitors. The antibody-coated beads were washed with PBS and incubated with U343 cell lysates. The lysate and beads were incubated overnight at 4 °C. The beads were separated using a magnetic rack and washed with 500 μL of cold nuclease-free 1×X PBS three times. RNA was extracted from the beads using the phenol: chloroform method as explained below. The RNA was analyzed by qPCR, and the level of Bcl-xL mRNA expression was measured.

Immunoprecipitation-Mass Spectrometry: Endo-IP was carried out as mentioned above. The final step of elution of proteins from the beads was carried out using an SP3 elution buffer (200 mM HEPES pH 8.0, 10% SDS, 200 mM DTT). The samples were sent to the British Columbia Cancer Research Centre’s proteomics core facility for mass spectrometry analysis. MS analysis was performed using the core facility’s well-established pipeline [47,48]. Briefly, experimental and control IPs were washed, alkylated, digested with trypsin, and labelled with one of 11 different isobaric tandem mass tags (TMT) [49], combined as a set of 11, and analyzed by liquid chromatography (LC)-MS/MS on an Orbitrap Fusion MS. Candidate interacting proteins were those with the highest significant (*p* < 0.05) enrichment scores in the experimental samples compared to the FBS control samples [47].

### 4.7. Western Blot Assay

The harvested cells were lysed in RIPA (50 mM Tris-HCl pH 7.4, 1 mM EDTA, 150 mM NaCl, 1% *v*/*v* NP 40, 0.5% *w*/*v* deoxycholic acid, 0.05% *w*/*v* SDS, protease and phosphatase inhibitors) and centrifuged at 10,000 *g* for 10 min to remove the cell debris. The protein concentration of the supernatant was quantified by the Bradford assay, using the 1X Bradford assay reagent from Bio-Rad, and according to the manufacturer’s protocol. Roughly 10–40 μg of total proteins from each lysate were separated on SDS-PAGE. The proteins were transferred onto a nitrocellulose membrane and incubated with 10% *w*/*v* fat-free milk in 1×X PBST (137 mM NaCl, 2.7 mM KCl, 10 mM Na_2_HPO_4_, 1.8 mM KH_2_PO_4_, and 1% tween 20) for 1 h followed by incubation with primary antibody overnight at 4 °C. The blots were incubated with a species-specific secondary antibody for one hour and visualized using an Amersham Imager 600. Between primary and secondary antibody probing, blots were washed with 0.1% 1×X PBST three times at 15 min intervals. The dilutions of antibodies used were as per the manufacturer’s recommendation. The list of antibodies used in the study can be found in Supplemental Table S2.

### 4.8. Puromycin Incorporation Assay

U343 cells were reverse-transfected with siRNA (siC, siPDCD4, and sieIF3F) as described in the section above. In the case of siRNA transfections, the time was optimized to 96 h before harvesting. An amount of 3 µg/mL of puromycin was added to the media 4 h before harvesting the cells. The cells were harvested and analyzed using the Western blot as mentioned in the above section.

### 4.9. Transformation

*Escherichia coli* (*E. coli*) strains such as DH5α (NEB), BL21 DE3 (NEB), BL21 ROSETTA 2 (DE3), PLYSS (Millipore Sigma), and TOP10 (Thermo Fischer Scientific) were used for transformation. DH5α and TOP10 were used for plasmid preparation, BL21 DE3 for PDCD4 and GST protein purification, and BL21 ROSETTA 2 (DE3) PLYSS for eIF3 subunit protein purification. An amount of 50 ng of plasmid DNA was added to the 25 μL aliquot of *E. coli* competent cells in a 1.5 mL tube. Cells were incubated on ice for 45 min, followed by a heat shock at 42 °C for 30 s. The cells were then placed on ice for 5 min and transferred into a culture tube containing 1 mL LB broth. The culture tube was incubated in a 37 °C shaker for 30 min. The cells were then streaked onto LB agar plates containing 100 μg/mL final concentration of the respective antibiotic and incubated overnight in a 37 °C incubator.

### 4.10. Recombinant Protein Purification

A single colony of *E. coli* containing His-PDCD4, GST-eIF3F, or GST bacterial expression construct was inoculated into a 3 mL LB broth (containing 100 μg/mL final concentration of ampicillin) and incubated overnight at 37 °C. Exactly 1 mL of the starter culture was inoculated into 200 mL of LB broth containing ampicillin and incubated at 37 °C with constant shaking at 150 rpm. The protein expression was induced with 0.5 mM IPTG at 0.6 OD of the *E. coli* cultures. The culture was incubated for 3 h after induction and then pelleted by centrifuging at 6000× *g* for 30 min. The pellet was suspended in 20 mL of lysis buffer (50 mM Tris-HCl pH 8, 1 M NaCl, 5% *v*/*v* glycerol, 0.5 mM PMSF, and 1 mM β-mercaptoethanol) and lysed using a French press. The lysate was centrifuged at 16,000× *g* for 30 min, and the supernatant was added to a 500 μL equilibrated Glutathione Sepharose 4 Fast Flow affinity medium (Cytiva, Burnaby, BC, Canada) or Ni-NTA agarose (Qiagen, Toronto, ON, Canada). The lysate was incubated with an affinity matrix for 1 h at 4 °C. The lysate was centrifuged at 1000× *g* for 2 min, and the supernatant was discarded. The matrix was washed with 20 mL of lysis buffer three times. In the case of His-PDCD4 protein purification, 40 mM of imidazole was added to the lysis buffer during the wash step. GST and GST-eIF3F were eluted using 10 mM glutathione in the lysis buffer. His-PDCD4 was eluted with 500 mM imidazole in the lysis buffer. Exactly 20 μL of each eluate was separated using SDS PAGE and visualized by Coomassie staining. The eluates were pooled based on purity and concentrated using an Amicon Ultra filtration device (Millipore Sigma) for in vitro assays. The Bradford assay quantified purified recombinant proteins.

### 4.11. In Vitro Protein Pull-Down Assay

Exactly 40 μL of Glutathione Sepharose 4 Fast Flow (Cytiva) affinity medium was equilibrated with 0.1 M TGEM buffer (20 mM Tris-HCl pH 7.9, 20% *v*/*v* glycerol, 1 mM EDTA, 5 mM MgCl_2_, 0.1% *v*/*v* NP 40, 1 mM DTT, 0.2 mM PMSF, 0.1 M NaCl). A total of 500 ng of the quantified bait protein (GST-eIF3F) was diluted to a final volume of 250 μL in 0.1 M TGEM buffer. The diluted sample was added to the equilibrated Glutathione Sepharose 4 Fast Flow affinity medium and incubated at 4 °C for 2 h. The matrix was then washed once with ice-cold 1 M TGEM buffer (20 mM Tris-HCl pH 7.9, 20% *v*/*v* glycerol, 1 mM EDTA, 5 mM MgCl_2_, 0.1% *v*/*v* NP 40, 1 mM DTT, 0.2 mM PMSF, 1 M NaCl) and twice with ice-cold 0.1 M TGMC (20 mM Tris-HCl pH 7.9, 20% *v*/*v* glycerol, 5 mM CaCl_2_, 5 mM MgCl_2_, 0.1% *v*/*v* NP 40, 1 mM DTT, 0.2 mM PMSF, 0.1 M NaCl) buffer to remove the unbound bait protein. An amount of 500 ng of PDCD4 was diluted to a final volume of 50 μL in 0.1 M TGMC buffer and added to the matrix coated with the bait protein. The prey protein was incubated with the bait protein at 4 °C for 2 h. The matrix was washed with 0.1 M TGEM buffer to remove the unbound prey protein. The proteins bound to the matrix were eluted by adding a 2×X concentration of Laemmli Sample Buffer and heated at 98 °C for 5 min. Exactly 50 ng of His-PDCD4 and eIF3F was loaded into 10% *w*/*v* SDS-PAGE and the pull-down samples as 10% input control. Samples were analyzed by Western blot.

### 4.12. Bicistronic Reporter Assay

The IRES containing bicistronic vector, pβGAL—Bcl-xL—IRES—CAT (pBcl-xL), and the control pβGAL—CAT (PBIC), were used as previously described [12,17]. The plasmids were transformed using an *E. coli* strain TOP10 (Thermo Scientific). The plasmids were then purified and transfected into U343 cells depleted with PDCD4/eIF3F/eIF3G using siRNAs. Post 48 h of siRNA transfection, the cells were transfected with 2 µg/mL of PBIC/pBcl-xL using Lipofectamine™ 2000. The cells were then harvested at 96 h as per the manufacturer’s protocol. The levels of βGAL and CAT were measured according to the protocol of the β-Gal Reporter Gene Assay kit (Roche, QC, Canada) and the CAT ELISA kit (Roche). The ratios of CAT/βGAL were then analyzed to calculate the relative IRES activity. Bicistronic reporter constructs (RLuc/FLuc) (Supplemental Table S1) were transfected to the PDCD4- and eIF3F-depleted U343 cells. Luciferase bicistronic reporter assays were carried out according to the manufacturer’s instructions using Luciferase Assay System (Promega, Madison, WI, USA).

### 4.13. Polysome Profiling

U343 cells were lysed from ten 10 cm plates using cold RNA lysis buffer (1.1 mL basic solution: 0.3 M NaCl, 15 mM MgCl_2_·6H_2_O, 15 mM Tris-HCl, pH 7.4, 110 µL 20% Triton X100, 2 µL SUPERase-In (Thermo Fischer), 5.5 µL of 1% cycloheximide (Sigma-Aldrich). Equal amounts of cell lysates were loaded on linear sucrose gradients ranging from 10% to 50%. A portion of the cell lysate was kept aside to isolate total RNA and to confirm the depletion of eIF3F or PDCD4 by Western blot analysis. A amount of 600 µL of cell lysate was loaded onto the sucrose gradients and centrifuged (SW41 rotor) at 39,000 rpm for 90 min at 4 °C. Gradients were then fractionated using a density gradient fractionation system (Brandel #SYN-202) at 1 mL each. Polysome profile was read using DATAQ software version 1.69. RNA from each fraction was isolated, and RT-qPCR was performed as explained below.

### 4.14. RNA Isolation

RNA isolation was done using the phenol-chloroform extraction method. Exactly 1 mL of polysome fraction or whole cell lysate was incubated with 50 µL of Proteinase K solution (57 µL 10% SDS, 7.5 µL 0.5 M EDTA, 1 µL Glycoblue, 4 µL of 20 mg/mL Proteinase K) at 55 °C for 1 h. Equal volumes of phenol: chloroform: isoamyl alcohol mixture (125:24:1, acidic pH 4.5) were added to each fraction/lysate. The fractions were centrifuged at 15,000 rpm for 5 min at room temperature. The aqueous phase was carefully removed, and an equal volume of chloroform was added. Centrifugation was repeated as mentioned above. To the aqueous phase, 1:10 volumes of 3 M sodium acetate (pH 5.2) and 1.5 volumes of chilled absolute ethanol were added and vortexed for ~15 s. The tubes were left overnight at −20 °C to precipitate. Overnight incubation was followed by centrifugation at 15,000× *g* for 30 min at 4 °C. The pellets were washed with chilled RNase-free 70% ethanol. Centrifugation was repeated as mentioned above. The pellets were air-dried and resuspended in RNase-free water. The quality of RNA was checked with a nanodrop (A260/280 ~1.7–2) and was further used for RT-qPCR analysis.

### 4.15. RT-qPCR

RNA was isolated as mentioned above and cDNA was generated from equal quantities of RNA using the qScript cDNA synthesis kit (Quanta Biosciences, Beverly, MA, USA). Quantitative PCR was performed in a CFX-96 real-time thermocycler (Bio-Rad) with PerfeCTa SYBR Green SuperMix (Quanta Biosciences) according to the manufacturer’s instructions. Negative controls without template DNA (NTR) were run in duplicate. Each reaction was run in duplicate with the following cycle conditions: 1 cycle at 95 °C for 3 min followed by 35 cycles at 95 °C for 15 s, the annealing temperature of 55 °C for 35 s, and 72 °C for 1 min. A melting curve step was added to examine the purity of the PCR product. This step consisted of a ramp of the temperature from 65 °C to 95 °C at an increment of 0.5 °C and a hold for 5 s at each step. Expression levels of, Bcl-xL, PDCD4, and eIF3F mRNA’s (relative to β-actin mRNA) were determined using the ΔΔC_t_ method. PDCD4, eIF3F, Bcl-xL, and Actin primers were obtained from Quantitect (Qiagen).

### 4.16. Electrophoretic Mobility Shift Assay (EMSA)

The sequence of interest (Bcl-xL IRES; 369 nucleotides) was amplified using Q5 High Fidelity polymerase (New England Biolabs, ON, Canada) according to the manufacturer’s instructions. The PCR product was purified using QIAquick^®^ Gel Extraction kit (Qiagen). Fluorescein-labelled RNA was obtained by in vitro transcription according to the labelling kit (Roche). According to the manufacturer’s instructions, fluorescein-12-UTP is incorporated by RNA polymerases at approximately every 20 to 25th nucleotide of the transcript under standard conditions. Amounts of 1.8 µM of eIF3F and 0.8 µM of PDCD4 (recombinantly purified proteins) were combined with 2.3 nM of labelled RNA in a Complex Assembly Buffer (150 mM NaCl, 20 mM HEPES, 1.5 mM MgCl_2_, 0.1 mM EDTA, 0.75 mM DTT, 10% Glycerol) and run on a 1% Native Agarose gel. Firstly, the RNA was heated to 65 °C for 3 min. The complex was then assembled and incubated on ice for 20 min before loading onto the gel. The gel was visualized on the Amersham Typhoon Imager. To calculate K_d_, a protein concentration range for eIF3F (0.4 µM, 0.8 µM, 1.2 µM, 1.8 µM, 2.0 µM, 2.6 µM) and PDCD4 (0.2 µM, 0.4 µM, 0.6 µM, 0.8 µM, 1.0 µM, 1.2 µM) was used. The K_d_ values were calculated and analyzed in GraphPad Prism 10 software.

### 4.17. Statistical Analysis

Unless specified, all quantified data show the mean ± standard error of the mean (SEM) for 3 biological replicates. An unpaired, two-tailed *t*-test determined statistical significance without assuming equal variance. The significance level was set at a *p*-value of 0.05. The significance is represented as nonsignificant = ns, *p* ≤ 0.05 = *, *p* ≤ 0.005 = **, *p* ≤ 0.001 = ***, and *p* ≤ 0.0001 = ****. Data was analyzed using GraphPad Prism, versions 9-10.

## Data Availability

The authors confirm that the data supporting the findings of this study are available within the article [or] its Supplementary Materials.

## Supplementary Materials

The following supporting information can be downloaded at: https://www.mdpi.com/article/10.3390/ijms27093955/s1, Supplemental Figures S1–S5. Supplemental Tables S1–S5 (IP-MS data, IRES bicistronic assay, and Luciferase assay, respectively) can be found on the following DOI/URL in a public repository, https://doi.org/10.5683/SP3/QJA2R5 and https://borealisdata.ca/dataset.xhtml?persistentId=doi:10.5683/SP3/QJA2R5 (accessed on 27 February 2026). References [17,20,28,50,51] are citied in the Supplementary Materials.
