# Supplementary material for: Eukaryotic Initiation Factor 3F (eIF3F) Regulates the IRES-Mediated Translation of Bcl-xL via Its Interaction with Programmed Cell Death 4 (PDCD4) Protein
Source: Int J Mol Sci. 2026 Apr 29;27(9):3955. doi: 10.3390/ijms27093955 (PMC13163806; doi:10.3390/ijms27093955)
Supplement: Supplementary file 1 [file ijms-27-03955-s001.zip › Supplemental Table 1_Constructs_with refs.pdf]

Supplemental Table S1

List of constructs, application of the constructs and their source.

Sl. No	Construct	Application	Source	Reference
1	2G-T.f	Bacterial expression of GST-eIF3F	Dr. Jamie H. D. Cate, University of California, Berkeley.	(Sun et al., 2011) [28]
2	His-PDCD4	Bacterial expression of His-PDCD4	Dr. Martin Holcik, CHEO Research Institute, University of Ottawa	(Liwak et al., 2012) [17]
3	pcDNA3-PDCD4-FLAG	Mammalian expression of FLAG-PDCD4		
4	pcDNA3-FLAG	Mammalian expression of FLAG-tag		
5	HA-eIF3F	Mammalian expression of HA-eIF3F	Dr. Nahum Sonenberg McGill University	(Martineau, Y., et al, MCB, 2014) [20]
6	βGAL-CAT (PBIC)	Bicistronic Reporter Assay	Dr. Martin Holcik, CHEO Research Institute, University of Ottawa	(Liwak et al., 2012) [17]
7	βGAL – Bcl-xL – IRES – CAT (pBcl-xL)	Bicistronic Reporter Assay	Sigma Millipore – Target sequence: CAGTCACAGATTGCACTCAAT	
8	sh87 (eIF3F) (# TRCN0000073987)	Polysome profiling		
9	Sh79 (PDCD4) (#TRCN0000059079)	Polysome profiling	Sigma Millipore – Target sequence: GCGGTTTGTAGAAGAATGTTT	
10	psPax2 (packaging plasmid)	Lentivirus production	Addgene -Plasmid #12260	Gift from Didier Trono
11	pMD2.G (envelope plasmid)	Lentivirus production	Addgene – Plasmid #12259	Gift from Didier Trono
12	pKS-FF-HCV-Ren	Luciferase Reporter Assay	Dr. Nahum Sonenberg McGill University	(Steinberger et al., 2020) [50]
13	CrPV	Luciferase Reporter Assay	Dr. Eric Jan University of British Columbia	(Qing S Wang, 2014) [51]
