# Supplementary material for: Eukaryotic Initiation Factor 3F (eIF3F) Regulates the IRES-Mediated Translation of Bcl-xL via Its Interaction with Programmed Cell Death 4 (PDCD4) Protein
Source: Int J Mol Sci. 2026 Apr 29;27(9):3955. doi: 10.3390/ijms27093955 (PMC13163806; doi:10.3390/ijms27093955)
Supplement: Supplementary file 1 [file ijms-27-03955-s001.zip › Supplemental Table 2_Antibodies 2.pdf]

Supplemental Table S2: The list of antibodies used and their source.

Antibody	Source
PDCD4	Rockland (#600-401-965), Proteintech (#12587-1-AP), Abcam (#ab80590)
PAIP	Abcam (#ab175211)
eIF3 subunits: eIF3B, eIF3D, eIF3E, eIF3F, eIF3G, eIF3H	**Abcam** eIF3B (#ab133601), eIF3D (#ab155419), eIF3E (#ab36766), eIF3F (#ab74568), eIF3G (#ab192601), eIF3H (#ab228536)
S6	Cell signaling technology (#2217)
pS6	Cell signaling technology (#4858S)
Bcl-xL (polyclonal and monoclonal)	Cell signaling technology (#2762S, #2764S)
XIAP (polyclonal and monoclonal)	Cell signaling technology (#2042S), Proteintech (#66800-1-Ig)
Puromycin	Sigma Millipore (#MABE343)
Actin	Bio-Rad (#12004163)
Anti-rabbit (secondary)	Abcam (#ab97051)
Anti-mouse (secondary)	Abcam (#ab6728)
Conformation-specific anti-rabbit	Cell signaling technology (#5127S)

Supplemental Table 3, 4, and 5 (IP-MS data, IRES bi-cistronic assay, and Luciferase assay, respectively) can be found on the following DOI/URL in a public repository.

https://doi.org/10.5683/SP3/QJA2R5

and

https://borealisdata.ca/dataset.xhtml?persistentId=doi:10.5683/SP3/QJA2R5
