# Supplementary material for: Eukaryotic Initiation Factor 3F (eIF3F) Regulates the IRES-Mediated Translation of Bcl-xL via Its Interaction with Programmed Cell Death 4 (PDCD4) Protein
Source: Int J Mol Sci. 2026 Apr 29;27(9):3955. doi: 10.3390/ijms27093955 (PMC13163806; doi:10.3390/ijms27093955)
Supplement: Supplementary file 1 [file ijms-27-03955-s001.zip › Supplementary Figures.pdf]

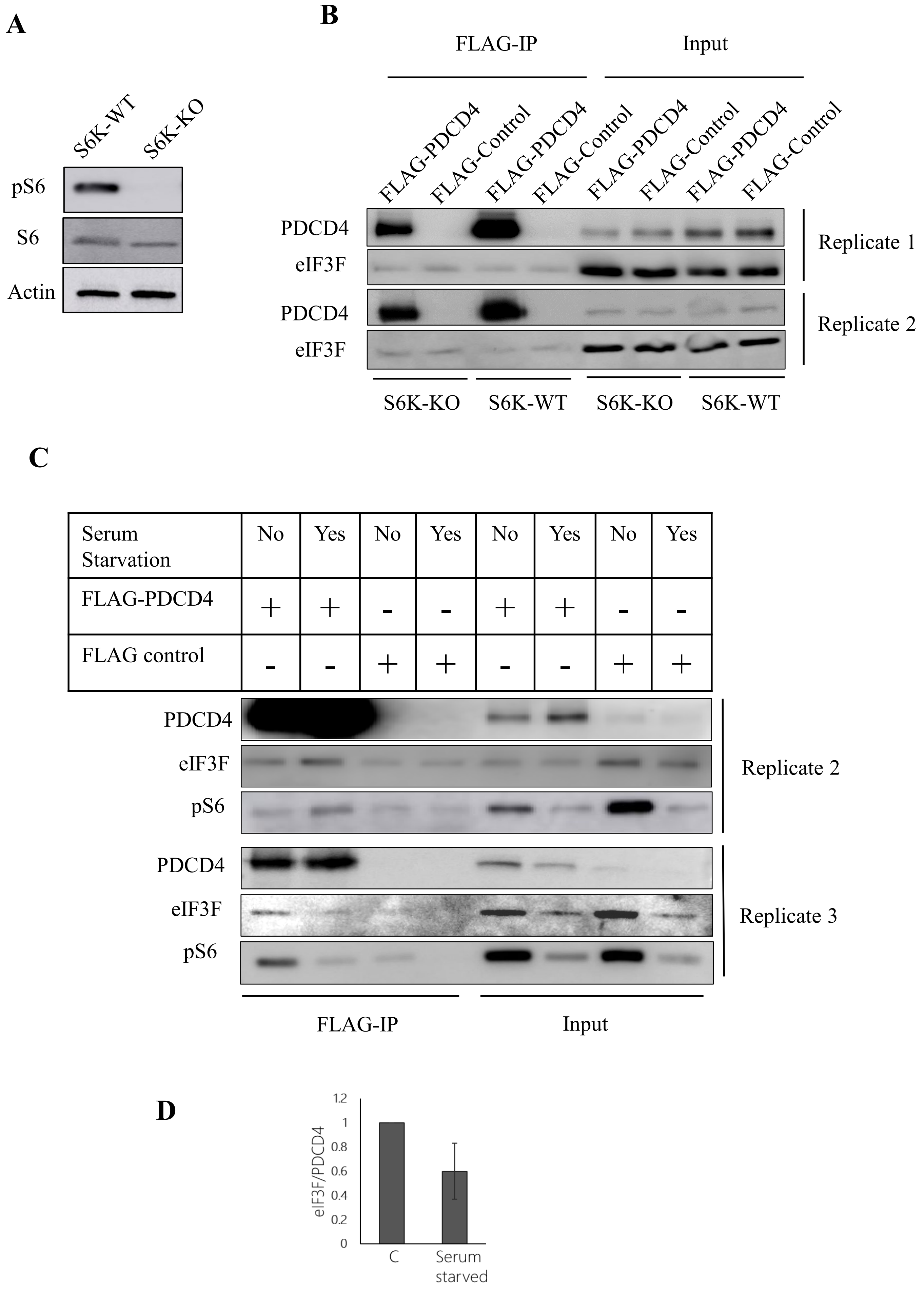
A
S6K-WT
S6K-KO
pS6
S6
Actin
B
FLAG-IP
Input
FLAG-PDCD4
FLAG-Control
FLAG-PDCD4
FLAG-Control
FLAG-PDCD4
FLAG-Control
FLAG-PDCD4
FLAG-Control
PDCD4
eIF3F
PDCD4
eIF3F
Replicate 1
Replicate 2
S6K-KO
S6K-WT
S6K-KO
S6K-WT
C
Serum Starvation
No
Yes
No
Yes
No
Yes
No
Yes
FLAG-PDCD4
+
+
-
-
+
+
-
-
FLAG control
-
-
+
+
-
-
+
+
PDCD4
eIF3F
pS6
Replicate 2
PDCD4
eIF3F
pS6
Replicate 3
FLAG-IP
Input
D
eIF3F/PDCD4
1.2
1
0.8
0.6
0.4
0.2
0
C
Serum starved

Supplementary Figure 1: S6 protein was equally expressed in wild-type and S6K double knockout MEFs. However, phosphorylation of S6 is not detected in S6K double knockout MEFs, which suggests the lack of S6K activity **(A)**. PDCD4 and eIF3F protein levels were similar in all four input lanes. In the co-IP lanes, eIF3F band intensity was similar in all four lanes, indicating no difference in interaction with PDCD4. This suggests that PDCD4 and eIF3 interaction is not affected in MEFs by the S6K activity **(B)**. Serum starvation decreased the S6 phosphorylation, which suggests decreased S6K activity **(C)**. eIF3F and pS6 were co-immunoprecipitated with FLAG-PDCD4. This interaction decreased with serum starvation, seen in the co-IP lanes **(C)**. However, the input lanes indicated a decrease in eIF3F protein levels in the total cell lysate **(D)**. This suggests that the PDCD4-eIF3F interaction is not affected by serum starvation.

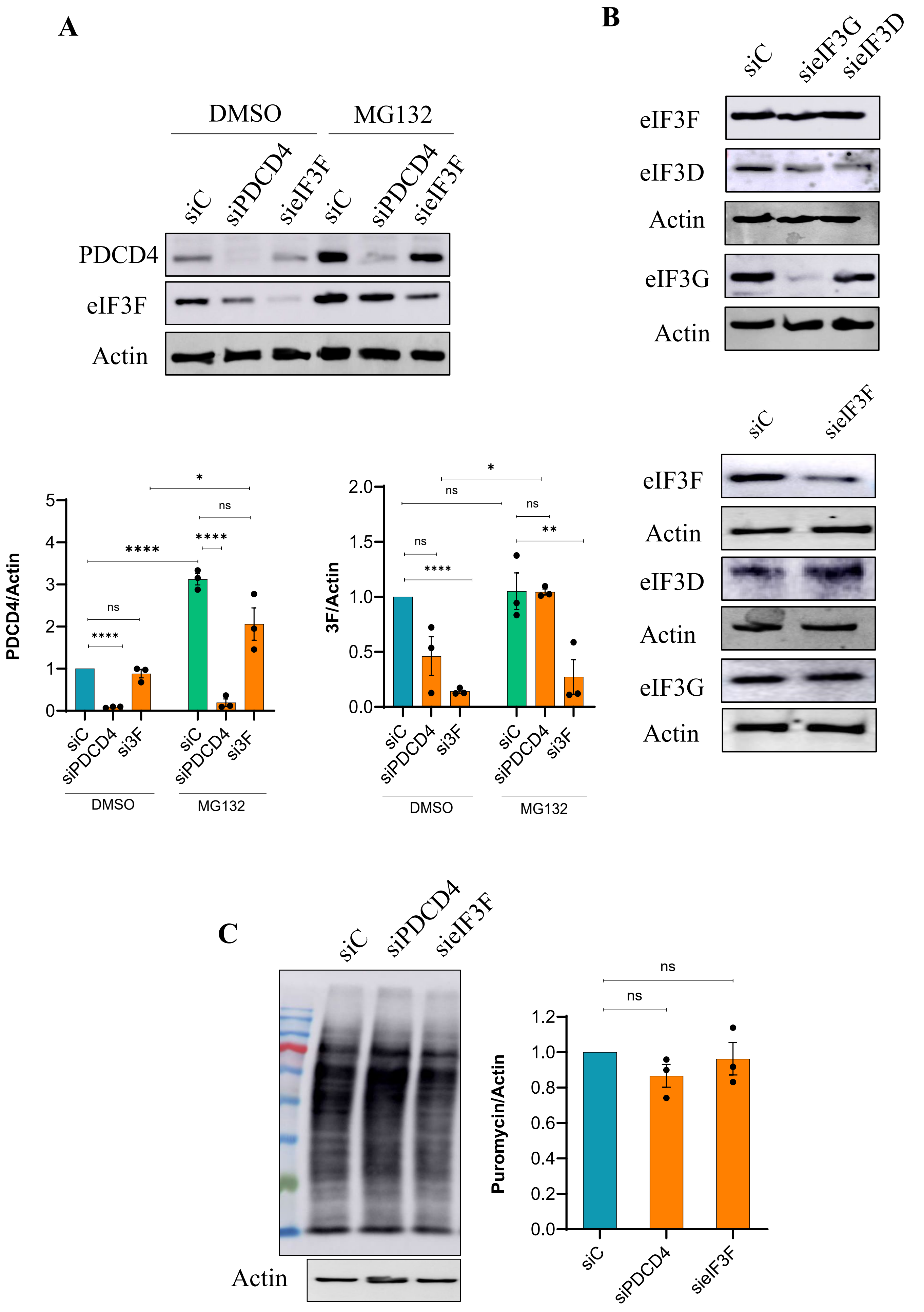
A
DMSO
MG132
siC
siPDCD4
sieIF3F
siC
siPDCD4
sieIF3F
PDCD4
eIF3F
Actin
B
siC
sieIF3G
sieIF3D
eIF3F
eIF3D
Actin
eIF3G
Actin
siC
sieIF3F
eIF3F
Actin
eIF3D
Actin
eIF3G
Actin
PDCD4/Actin
0
1
2
3
4
5
****
ns
****
****
ns
*
siC
siPDCD4
si3F
siC
siPDCD4
si3F
DMSO
MG132
3F/Actin
0.0
0.5
1.0
1.5
2.0
ns
****
ns
*
ns
**
siC
siPDCD4
si3F
siC
siPDCD4
si3F
DMSO
MG132
C
siC
siPDCD4
sieIF3F
Actin
Puromycin/Actin
0.0
0.2
0.4
0.6
0.8
1.0
1.2
ns
ns
siC
siPDCD4
sieIF3F

Supplementary Figure 2: Treatment of MG132 (a potent proteasomal inhibitor) prevented the degradation of PDCD4 in eIF3F-depleted U343 cells. Likewise, MG132 treatment also prevented the degradation of eIF3F in PDCD4-depleted U343 cells **(A)**. In a control experiment of a single biological replicate, eIF3G, eIF3D, or eIF3F were depleted from U343 cells, and the levels of PDCD4, eIF3F, and Bcl-xL were determined. Upon eIF3G depletion, the levels of PDCD4 were reduced. However, the levels of eIF3F and Bcl-xL were not affected. Under eIF3D depletion, the levels of PDCD4 were not significantly affected, however, the levels of eIF3F and Bcl-xL did not change significantly **(B; top panel)**. We also show that the levels of eIF3D and eIF3G did not change under eIF3F depletion **(B; bottom panel).** Puromycin Incorporation Assay showed that under PDCD4 and eIF3F depletion, the overall translation was not affected **(C)**.

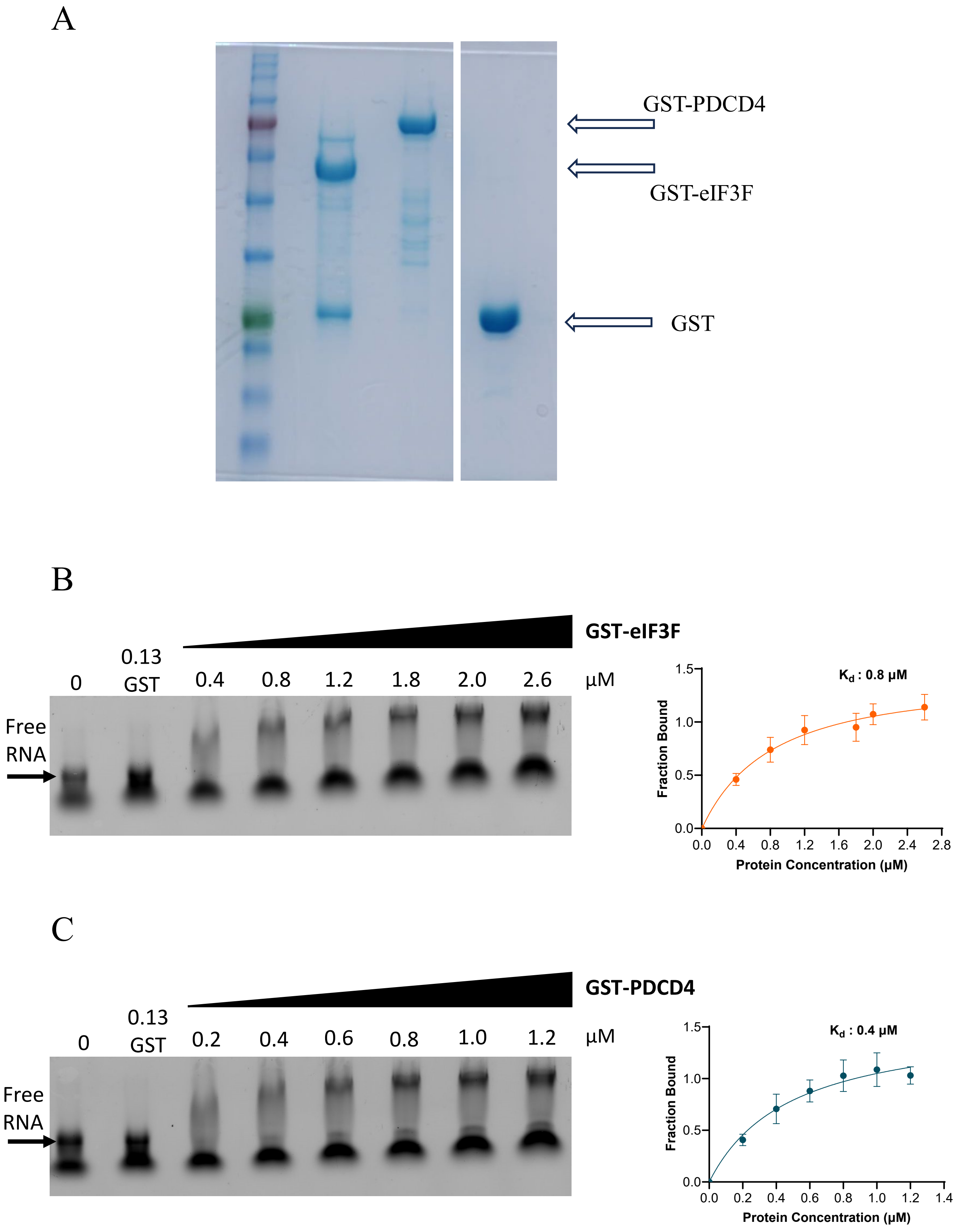

Supplementary Figure 3: GST-PDCD4, GST-eIF3F and GST proteins were purified to homogeneity for EMSA **(A).** Optimization of protein concentration and K_d values of proteins for EMSA. Both PDCD4 and eIF3F proteins were optimized at 0.8 µM and 1.8 µM for the EMSA gels, respectively. Final concentration of RNA was 2.3 nM **(B and C)**

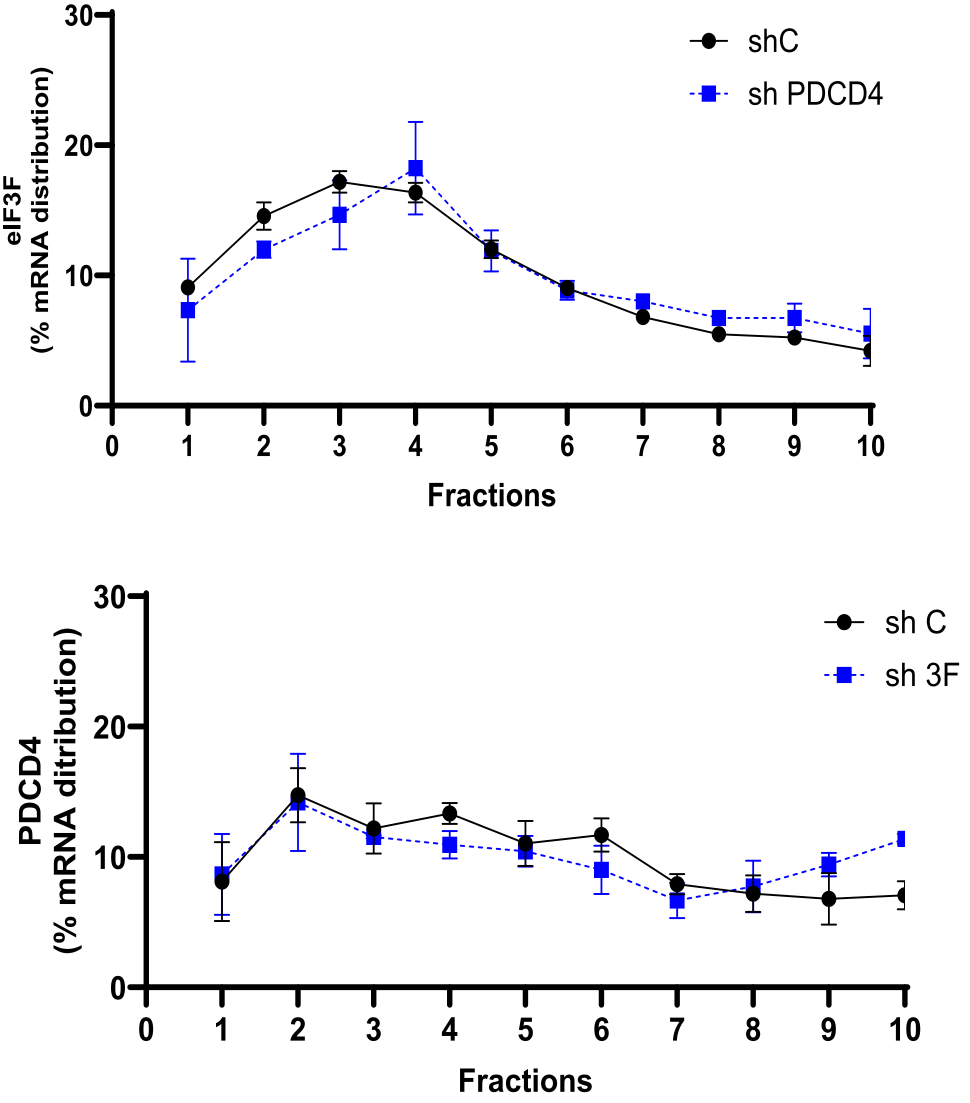

Supplementary Figure 4: The level of translation of eIF3F did not change significantly in shPDCD4 condition compared to shC. The level of translation of PDCD4 did not show any difference in sheIF3F condition compared to shC.

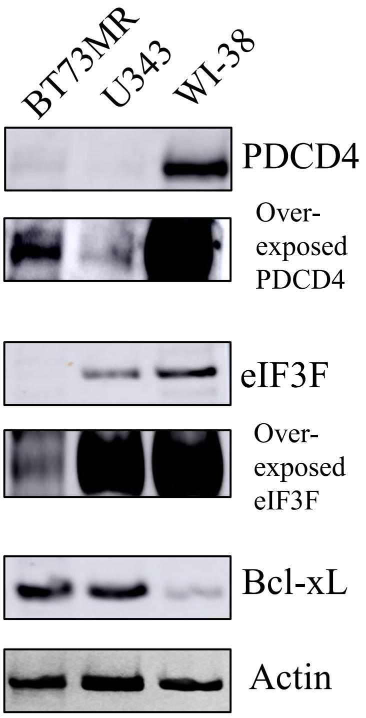

Supplementary Figure 5: Relative levels of PDCD4, eIF3F, and Bcl-xL in GBM lines (BT73MR and U343) and WI-38 fibroblasts. Over-exposed bands were also visualized to detect the presence of PDCD4 and eIF3F in GBM lines.
